# Analysis of Side-band Inequivalence

**DOI:** 10.1038/s41598-019-45580-7

**Published:** 2019-06-24

**Authors:** Sina Khorasani

**Affiliations:** Vienna Center for Quantum Science and Technology, Boltzmanngasse 5, 1090 Vienna, Austria

**Keywords:** Theoretical physics, Quantum optics

## Abstract

Frequency shifts of red- and blue-scattered (Stokes/anti-Stokes) side-bands in quantum optomechanics are shown to be counter-intuitively inequal, resulting in an unexpected symmetry breaking. This difference is referred to as Side-band Inequivalenve (SI), which normally leans towards red, and being a nonlinear effect it depends on optical power or intracavity photon number. Also there exists a maximum attainable SI at an optimal operation point. The mathematical method employed here is a combination of operator algebra equipped with harmonic balance, which allows a clear understanding of the associated nonlinear process. This reveals the existence of three distinct operation regimes in terms of pump power, two of which have immeasurably small SI. Compelling evidence from various experiments sharing similar interaction Hamiltonians, including quantum optomechanics, ion/Paul traps, electrooptic modulation, Brillouin scattering, and Raman scattering unambiguously confirm existence of a previously unnoticed SI.

## Introduction

The nonlinearity of optomechanical interaction^1–10^ causes scattering of incident photons with the annihilator $\hat{a}$ from the cavity unto either red- or blue-shifted photons through annihilation or generation of a cavity phonon with the annihilator $\hat{b}$, giving rise to the first-order mechanical side-bands. Taking the optical frequency *ω* to be at a detuning Δ = *ω*_c_ − *ω* from cavity resonance *ω*_c_, the *ν*− th order sidebands are naturally expected to occur at the detunings ${{\rm{\Delta }}}_{\pm \nu }={\rm{\Delta }}\mp \nu {\rm{\Omega }}$, where Ω represents the mechanical frequency. As a results, the first-order mechanical side-bands of scattered red Δ_+1_ and blue Δ_−1_ processes must average out back to the original pump detuning Δ.

**Figure 1:**
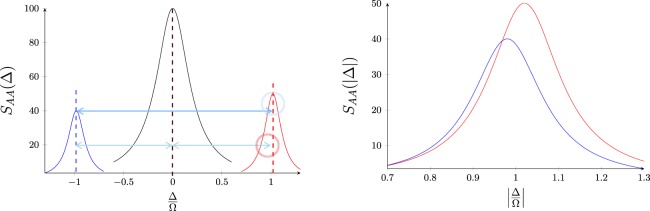
Illustration of two basic symmetry breakings in a hypothetical quantum optomechanical cavity at resonant pump, and in absence of cooling tone: (left) separated side-bands in a heterodyne setup; (right) zoom out of overlapping side-bands in a homodyne detection around mechanical frequency with |Δ| = Ω. The red detuning exceeds that of blue detuning, shown in pale red circle, corresponding to the side-band inequivalence (pale red circle), and also the height of red peak exceeds that of blue peak, corresponding to side-band asymmetry (pale blue circle). Here, *S*_AA_(Δ) represents the spectral noise density. Blue and red Lorentzian linewidths are 0.1Ω, while the central resonance has a linewidth of 0.2Ω. Side-band Inequivalence is chosen to be $\bar{\delta }=\delta /{\rm{\Omega }}=0.02$ towards red side-band.

Defining the deviation of this average from Δ, as $\delta =\frac{1}{2}({{\rm{\Delta }}}_{+1}+{{\rm{\Delta }}}_{-1})-{\rm{\Delta }}$, then one may normally expect *δ* = 0. A non-zero *δ* would have otherwise implied the so-called Side-band Inequivalence (SI). This type of asymmetry appears to have a classical nonlinear nature, and is illustrated in Fig. 1.

There is, however, another well-known type of side-band asymmetry between the red and blue side-bands in the context of optomechanics, which has a quantum nature and may be used for instance to accurately determine the absolute temperature through a reference-free optomechanical measurement^11,12^. This is based on the ratio of Stokes to anti-Stokes Raman transition rates, which is equal to exp(*ℏ*Ω/*k*_B_*T*) where *k*_B_ is the Boltzmann’s constant and *T* is the absolute temperature^13,14^. Also illustrated in Fig. 1, clearly, SI is quite different from this type of side-band asymmetry.

While both time-reversal symmetry and energy conservation are fundamentally preserved in this scattering process, a nonlinear analysis of quantum optomechanics using the recently developed method of higher-order operators^15–19^ necessitates a slight difference among detunings of blue and red-scattered photons, the amount of which was initially found to increase roughly proportional to the intracavity photon number $\bar{n}$. Here, $\bar{n}$ is defined as the steady-state mean-value of the number operator $\hat{n}={\hat{a}}^{\dagger }\hat{a}$.

Surprisingly enough, this disagreement satisfying *δ* ≠ 0 does not violate the energy conservation law, actually allowed by the finite cavity linewidth as well as the single-photon/single-phonon nature of the process involved. Moreover, the time-reversal symmetry is also preserved.

Among the pool of available experimental data, only a handful of side-band resolved cavities reveal this disagreement^15^. Some initial trial experiments recently done at extremely high intracavity photon numbers $\bar{n}$, and/or extremely large single-photon optomechanical interaction rates *g*_0_, though, failed to demonstrate its existence. This may raise the speculation that whether SI would have been merely a mathematical artifact, or something has been missing due to not doing the operator analysis to the highest-order.

A careful analysis of this phenomenon, however, confirms the latter, thus classifying the quantum optomechanical interaction into three distinct regimes with different behaviors:

- Fully Linear: This regime can be investigated using the lowest-order analysis and first-order operators, which is conventionally done by linearizing the Hamiltonian around equilibrium points. This will require the four-dimensional basis of first-order ladder operators $\{\hat{a},{\hat{a}}^{\dagger },\hat{b},{\hat{b}}^{\dagger }\}$ and is indeed quite sufficient to understand many of the complex quantum optomechanical phenomena^20^.
- Weakly Nonlinear: This regime requires higher-order operator analysis of at least second-order. This can be done using the three-dimensional reduced basis^15,20^ given as $\{\hat{a},\hat{a}\hat{b},\hat{a}{\hat{b}}^{\dagger }\}$.
- Strongly Nonlinear: Full understanding of this interaction regime requires the highest-order analysis using third-order operators. Referred as to the minimal basis^15,20^, the convenient reduced choice is the two-dimensional basis $\{{\hat{n}}^{2},\hat{n}\hat{b}\}$.

In the latter regime, the behavior of governing equations is in such a way that a fully-linearized analysis of fluctuations mostly happens to work. This definitely sounds strange, unless we note that for the strongly non-linear regime, the optical pump level is so strong that the quantumness of intracavity photons disappear and we can do the replacements $\hat{n}(t)\to \bar{n}(t)$ and $\hat{a}(t)\to {e}^{i{\rm{\Delta }}t}{\rm{a}}(t)$. Once the steady-state is reached, the minimal basis reduces to ${\bar{n}}^{2},\bar{n}\hat{b}$. It is easy to see that $\bar{n}$ stays almost constant so that mechanics is still quantized and expressed via $\hat{b}$. Based on the Langevin’s equations for classical electromagnetic field a(*t*) and quantized mechanics $\hat{b}(t)$ it is easy to see that the only surviving frequencies are ${\rm{\Delta }}\pm {\rm{\Omega }}\pm 2{\rm{\Omega }}\pm \cdots$. Hence, quantumness of optics is absolutely necessary for the SI to occur, and this disappears when the optical pump is so strong that the electromagnetic field can be treated classically.

Side-band Inequivalence is essentially forbidden in the fully linear regime, and it also quickly fades away in the strongly nonlinear regime. But it may only happen in the weakly nonlinear regime. This is now also confirmed both by the higher-order operator method and extensive calculations. It typically does not exceed one part in million to one part in ten thousand in solid state quantum optomechanics, and therefore, it is a very delicate phenomenon and elusive to observe. As it will be shown, other experiments such as Raman scattering may instead exhibit much stronger inequivalences.

This letter provides a direct route towards clear understanding of this complex nonlinear phenomenon. Using a combination of operator algebra and harmonic balance (used in analysis of laser diodes)^21^, we obtain a closed form expression for SI *δ* as a function of intracavity photon number $\bar{n}$, which is expected to be valid through all three above operation regimes, and for any arbitrarily chosen set of optomechanical parameters. Not only the findings of this work reproduce the approximate linear expression found earlier through second-order operators^15^ for the weakly nonlinear regime, but also we can show that there is an optimal point at which the SI attains a maximum. Moving away from the optimal point, both at the much smaller and much larger pump rates, *δ* attains much smaller values, tending to zero in the limit of very large $\bar{n}$. An analytical treatment of nonlinear optomechanical equations based on breathing solutions is also presented, which again confirms the existence of inequivalence towards red and is presented in the Supplementary Information.

This will be greatly helpful to designate the investigation range of experimental parameters given any available optomechanical cavity. Furthermore, it marks a clear and definable border among the three above-mentioned operation regimes.

## Results

The analysis of SI proceeds with considering the behavior of optomechanical cavity under steady-state conditions. We will focus only on the first-order side-bands and discard all other contributions coming from or to the second- and higher-order side-bands. We consider a single-frequency pump with ideally zero linewidth at a given detuning Δ, which normally gives rise to two stable blue- and red- side-bands. Hence, the time-dependence of the photon annihilator will look like

1 $$\hat{a}(t)={\hat{a}}_{0}{e}^{i{\rm{\Delta }}t}+{\hat{a}}_{b}{e}^{i({\rm{\Delta }}-{\rm{\Omega }}+\frac{1}{2}\delta )t}+{\hat{a}}_{r}{e}^{i({\rm{\Delta }}+{\rm{\Omega }}+\frac{1}{2}\delta )t}+\cdots ,$$

where ${\hat{a}}_{0}$, ${\hat{a}}_{b}$, and ${\hat{a}}_{r}$ respectively correspond to the central excitation resonance at pump frequency, and blue- and red-detuned side-bands. In the weak coupling regime where $g={g}_{0}\sqrt{\bar{n}}\ll {\rm{\Omega }}$, the *n* + 1− th order side-band is typically (*g*/Ω)^*n*^ times weaker in amplitude than the 1st-order side-band. This means, for instance, that the second-order side-band is *g*/Ω weaker than the first-order side-bands. In that sense, their respective contributions and significance diminish very rapidly with their growing order. So, there is good reason to agree that the truncation in (1) up to the first-order side-bands is a rather acceptable approximation. Inclusion of 2nd-order terms such as ${\hat{a}}_{bb}{e}^{i({\rm{\Delta }}-2{\rm{\Omega }}+\delta )t}$ and ${\hat{a}}_{rr}{e}^{i({\rm{\Delta }}+2{\rm{\Omega }}+\delta )t}$, will result in minor modification of following expressions. It has to be noticed that the red and blue time dependences of *n*− th order processes must be ${e}^{i({\rm{\Delta }}-n{\rm{\Omega }}+\frac{n}{2}\delta )t}$ and ${e}^{i({\rm{\Delta }}-n{\rm{\Omega }}+\frac{n}{2}\delta )t}$, showing that the associated SI with the *n*− th order side-bands should be *nδ*.

We here furthermore have ignored the symmetrical movement of side-bands which should correspond to the optomechanical spring effect *δ*Ω^1,3^, but this correction is zero at Δ = 0 while *δ* is independent of Δ (in Supplementary Information we show that contribution of this term is insignificant). It is the asymmetric movements of side-bands which gives rise to SI. In order to do so, it would have been enough to take the ansatz $\hat{a}(t)={\hat{a}}_{0}{e}^{i{\rm{\Delta }}t}+{\hat{a}}_{b}{e}^{i({\rm{\Delta }}-\varpi +\frac{1}{2}\delta )t}+{\hat{a}}_{r}{e}^{i({\rm{\Delta }}+\varpi +\frac{1}{2}\delta )t}+\cdots$ instead, with *ϖ* = Ω + *δ*Ω being the shifted mechanical frequency due to the optomechanical spring effect.

The steady-state time-average of central excitation satisfies ${\hat{a}}_{0}=\sqrt{\bar{n}}$, where $\bar{n}$ can be determined by solution of a third-order algebraic equation once the optical power pump rate *P*_op_, detuning Δ, external coupling *η* and all other optomechanical parameters are known. The standard set of basic optomechanical parameters needed here are mechanical frequency Ω, optical decay rate *κ*, and mechanical decay rate Γ. Therefore, the photon number operator up to the first side-bands will behave as

2 $$\begin{array}{rcl}\hat{n}(t) & = & {\hat{a}}_{0}^{\dagger }{\hat{a}}_{0}+{\hat{a}}_{b}^{\dagger }{\hat{a}}_{b}+{\hat{a}}_{r}^{\dagger }{\hat{a}}_{r}+{\hat{a}}_{b}^{\dagger }{\hat{a}}_{0}{e}^{-i(-{\rm{\Omega }}+\frac{1}{2}\delta )t}+{\hat{a}}_{0}^{\dagger }{\hat{a}}_{b}{e}^{i(-{\rm{\Omega }}+\frac{1}{2}\delta )t}\\  &  & +\,{\hat{a}}_{r}^{\dagger }{\hat{a}}_{0}{e}^{-i({\rm{\Omega }}+\frac{1}{2}\delta )t}+{\hat{a}}_{0}^{\dagger }{\hat{a}}_{r}{e}^{i({\rm{\Omega }}+\frac{1}{2}\delta )t}+\cdots ,\end{array}$$

while the mechanical annihilator will exhibit a closely spaced doublet around the mechanical frequency spaced within *δ* as

3 $$\hat{b}(t)={\hat{b}}_{0}+{\hat{b}}_{b}{e}^{-i({\rm{\Omega }}-\frac{1}{2}\delta )t}+{\hat{b}}_{r}{e}^{-i({\rm{\Omega }}+\frac{1}{2}\delta )t}+\cdots .$$

Inclusion of 2nd-order side-bands would have generated extra terms in (2) such as ${\hat{a}}_{bb}^{\dagger }{\hat{a}}_{r}$ and ${\hat{a}}_{rr}^{\dagger }{\hat{a}}_{b}$ and their conjugates as well as ${\hat{a}}_{bb}^{\dagger }{\hat{a}}_{bb}+{\hat{a}}_{rr}^{\dagger }{\hat{a}}_{rr}$. For instance, ${\hat{a}}_{bb}^{\dagger }{\hat{a}}_{r}$ should have added up to ${\hat{a}}_{b}^{\dagger }{\hat{a}}_{0}$ and so on. These ignored terms all would cause third- or fourth-order corrections to (2), which obviously were dropped.

Here, the average mechanical displacement satisfies

4 $${b}_{0}=\langle {\hat{b}}_{0}\rangle =\frac{i{g}_{0}\bar{n}}{i{\rm{\Omega }}+\frac{1}{2}{\rm{\Gamma }}}.$$

Now, let us get back to the Langevin equation for mechanical motions, which simply is

5 $$\frac{d}{dt}\hat{b}(t)=(-i{\rm{\Omega }}-\frac{1}{2}{\rm{\Gamma }})\hat{b}(t)-i{g}_{0}\hat{n}(t)+\sqrt{{\rm{\Gamma }}}{\hat{b}}_{{\rm{in}}}(t),$$

where ${\hat{b}}_{{\rm{in}}}(t)$ is the operator for mechanical fluctuations. For the purpose of our analysis here, all fluctuations can be discarded since they are irrelevant to the formation of side-band frequencies and average out to zero. Using (2) and (3) we get

6 $$\begin{array}{c}-i({\rm{\Omega }}+\frac{\delta }{2}){\hat{b}}_{r}{e}^{-i({\rm{\Omega }}+\frac{\delta }{2})t}-i({\rm{\Omega }}-\frac{\delta }{2}){\hat{b}}_{b}{e}^{-i({\rm{\Omega }}-\frac{\delta }{2})t}\approx -(i{\rm{\Omega }}+\frac{{\rm{\Gamma }}}{2}){\hat{b}}_{r}{e}^{-i({\rm{\Omega }}+\frac{\delta }{2})t}\\ -(i{\rm{\Omega }}+\frac{{\rm{\Gamma }}}{2}){\hat{b}}_{b}{e}^{-i({\rm{\Omega }}-\frac{\delta }{2})t}-i{g}_{0}{\hat{a}}_{0}^{\dagger }{\hat{a}}_{b}{e}^{-i({\rm{\Omega }}-\frac{\delta }{2})t}-i{g}_{0}{\hat{a}}_{r}^{\dagger }{\hat{a}}_{0}{e}^{-i({\rm{\Omega }}+\frac{\delta }{2})t}+\cdots .\end{array}$$

From the above, we obtain two key operator equations

7 $$\begin{array}{rcl}{\hat{b}}_{r} & = & \frac{-i2{g}_{0}}{-i\delta +{\rm{\Gamma }}}{\hat{a}}_{r}^{\dagger }{\hat{a}}_{0},\\ {\hat{b}}_{b} & = & \frac{-i2{g}_{0}}{i\delta +{\rm{\Gamma }}}{\hat{a}}_{0}^{\dagger }{\hat{a}}_{b}.\end{array}$$

In a similar manner, the Langevin equation for the photon annihilator is

8 $$\frac{d}{dt}\hat{a}(t)=(i{\rm{\Delta }}-\frac{1}{2}\kappa )\hat{a}(t)+i{g}_{0}\hat{a}(t)[\hat{b}(t)+{\hat{b}}^{\dagger }(t)]+\sqrt{\kappa }{\hat{a}}_{{\rm{in}}}.$$

Using (1) and (3) we obtain

9 $$\begin{array}{c}i{\rm{\Delta }}{\hat{a}}_{0}{e}^{i{\rm{\Delta }}t}+i({\rm{\Delta }}-{\rm{\Omega }}+\frac{\delta }{2}){\hat{a}}_{b}{e}^{i({\rm{\Delta }}-{\rm{\Omega }}+\frac{\delta }{2})t}+i({\rm{\Delta }}+{\rm{\Omega }}+\frac{\delta }{2}){\hat{a}}_{r}{e}^{i({\rm{\Delta }}+{\rm{\Omega }}+\frac{\delta }{2})t}\\ \approx \,(i{\rm{\Delta }}-\frac{\kappa }{2})[{\hat{a}}_{0}{e}^{i{\rm{\Delta }}t}+{\hat{a}}_{b}{e}^{i({\rm{\Delta }}-{\rm{\Omega }}+\frac{\delta }{2})t}+{\hat{a}}_{r}{e}^{i({\rm{\Delta }}+{\rm{\Omega }}+\frac{\delta }{2})t}]+i{g}_{0}[{\hat{a}}_{0}{e}^{i{\rm{\Delta }}t}+{\hat{a}}_{b}{e}^{i({\rm{\Delta }}-{\rm{\Omega }}+\frac{\delta }{2})t}\\ \,+\,{\hat{a}}_{r}{e}^{i({\rm{\Delta }}+{\rm{\Omega }}+\frac{\delta }{2})t}][{\hat{x}}_{0}+{\hat{b}}_{r}{e}^{-i({\rm{\Omega }}+\frac{\delta }{2})t}+{\hat{b}}_{b}{e}^{-i({\rm{\Omega }}-\frac{\delta }{2})t}+{\hat{b}}_{r}^{\dagger }{e}^{i({\rm{\Omega }}+\frac{\delta }{2})t}+{\hat{b}}_{b}^{\dagger }{e}^{i({\rm{\Omega }}-\frac{\delta }{2})t}].\end{array}$$

where ${\hat{x}}_{0}={\hat{b}}_{0}+{\hat{b}}_{0}^{\dagger }$. This will yield the further operator equations as

10 $$\begin{array}{rcl}{\frac{\kappa }{2i{g}_{0}}{\hat{a}}_{0}} & = &  {{\hat{a}}_{0}{\hat{x}}_{0}+{\hat{a}}_{b}{\hat{b}}_{b}^{\dagger }+{\hat{a}}_{r}{\hat{b}}_{r},}\\{[\frac{i(-{\rm{\Omega }}+\frac{\delta }{2})+\frac{\kappa }{2}}{i{g}_{0}}]{\hat{a}}_{b} } & = & {\hat{a}_{b}{\hat{x}}_{0}+{\hat{a}}_{0}{\hat{b}}_{b},}\\{[\frac{i({\rm{\Omega }}+\frac{\delta }{2})+\frac{\kappa }{2}}{i{g}_{0}}]{\hat{a}}_{r} } & = &  {{\hat{a}}_{r}{\hat{x}}_{0}+{\hat{a}}_{0}{\hat{b}}_{r}^{\dagger }.}\end{array}$$

Now, substituting whatever we have in hand in the second equation of (10), and taking expectation values at the end, we obtain a key algebraic equation in terms of *δ* as

11 $$i(-{\rm{\Omega }}+\frac{1}{2}\delta )+\frac{1}{2}\kappa =i{g}_{0}{x}_{0}-i{g}_{0}\sqrt{\bar{n}}\frac{2i{g}_{0}\sqrt{\bar{n}}}{i\delta +{\rm{\Gamma }}}.$$

with ${x}_{0}={b}_{0}+{b}_{0}^{\ast }$. Rearrangement of the above gives rise to the equation

12 $${\delta }^{2}-[2{\rm{\Omega }}+i\gamma +2{g}_{0}{x}_{0}]\delta +[(2i{\rm{\Omega }}-\kappa ){\rm{\Gamma }}+4{g}_{0}^{2}\bar{n}+2i{\rm{\Gamma }}{g}_{0}{x}_{0}]=0,$$

in which ${x}_{0}={b}_{0}+{b}_{0}^{\ast }$ and *γ* = *κ* + Γ is the total optomechanical decay rate^15,17^.

This approximate nature of this equation will yield complex values for *δ* the imaginary value of which has to be discarded. In practice, we observe that imaginary values are much smaller than real values for practical choices of optomechanical parameters. Furthermore, it leaves room to ignore the square terms *δ*^2^, to admit the solution

13 $$\delta =\Re [\frac{(2i{\rm{\Omega }}-\kappa ){\rm{\Gamma }}+4{g}_{0}^{2}\bar{n}+2i{\rm{\Gamma }}{g}_{0}{x}_{0}}{2{\rm{\Omega }}+i\gamma +2{g}_{0}{x}_{0}}].$$

This solution can be put into the more convenient form using (4) and further simplification as

14 $$\delta (\bar{n})=\Re [\frac{A+B\bar{n}}{C-iD\bar{n}}]=\frac{\Re [A{C}^{\ast }]+(B\Re [C]-\Im [A]D)\bar{n}}{|C{|}^{2}-2\Im [C]D\bar{n}+{D}^{2}{\bar{n}}^{2}}=\frac{[2{{\rm{\Gamma }}}^{2}{\rm{\Omega }}]+2{\rm{\Omega }}(B-{\rm{\Gamma }}D)\bar{n}}{|C{|}^{2}-4{\rm{\Omega }}D\bar{n}+{D}^{2}{\bar{n}}^{2}},$$

where

15 $$\begin{array}{rcl}A & = & {\rm{\Gamma }}(2i{\rm{\Omega }}-\kappa )+\frac{4{g}_{0}^{2}\bar{n}(i+1){\rm{\Gamma }}{\rm{\Omega }}}{{{\rm{\Omega }}}^{2}+\frac{1}{4}{{\rm{\Gamma }}}^{2}},\\ B & = & \frac{4{g}_{0}^{2}{({\rm{\Omega }}-\frac{1}{2}{\rm{\Gamma }})}^{2}}{{{\rm{\Omega }}}^{2}+\frac{1}{4}{{\rm{\Gamma }}}^{2}}={B}^{\ast },\\ C & = & i\gamma +2{\rm{\Omega }},\\ D & = & \frac{4{g}_{0}^{2}{\rm{\Omega }}}{{{\rm{\Omega }}}^{2}+\frac{1}{4}{{\rm{\Gamma }}}^{2}}={D}^{\ast }.\end{array}$$

In a first approximation, *A *≈ Γ(2*i*Ω − *κ*) can be used. Further simplifications are shown and discussed in the following.

### Limiting cases

The expression (14) obtained for the SI has interesting properties at the limits of zero and infinite intracavity photon number. We may obtain here after some simplification easily the asymptotic expressions

16 $$\begin{array}{lll}\mathop{\mathrm{lim}}\limits_{\bar{n}\to \infty }\delta (\bar{n}) &  \sim  & \frac{{\rm{\Omega }}}{\beta \bar{n}},\\ \mathop{\mathrm{lim}}\limits_{\bar{n}\to 0}\delta (\bar{n}) &  \sim  & \frac{2{{\rm{\Gamma }}}^{2}{\rm{\Omega }}}{4{{\rm{\Omega }}}^{2}+{\gamma }^{2}}\approx 0,\end{array}$$

where $\beta =2{g}_{0}^{2}/{{\rm{\Omega }}}^{2}$, while noting that Γ ≪ Ω and also for a side-band resolved cavity *κ* ≪ Ω, together which we have *κ* < *γ*  ≪ Ω.

One should take into account the fact that for Doppler cavities, side-bands normally resolve well enough for a decisive measurement^20^, and the concept of SI is only practically meaningful for side-band resolved cavities. Therefore, the following approximations are valid

17 $$\begin{array}{rcl}A & \approx  & 2i{\rm{\Gamma }}{\rm{\Omega }},\\ B & \approx  & 4{g}_{0}^{2},\\ C & \approx  & 2{\rm{\Omega }},\\ D & \approx  & \frac{4{g}_{0}^{2}}{{\rm{\Omega }}},\\ \delta (\bar{n}) & \approx  & \frac{2{{\rm{\Gamma }}}^{2}{\rm{\Omega }}+8{g}_{0}^{2}{\rm{\Omega }}\bar{n}}{{\gamma }^{2}+4{{\rm{\Omega }}}^{2}{[1-2{({g}_{0}/{\rm{\Omega }})}^{2}\bar{n}]}^{2}}.\end{array}$$

As long as satisfies $\bar{n} <  < 2|\Im [C]|/D={{\rm{\Omega }}}^{2}/{g}_{0}^{2}$, then second order term ${\bar{n}}^{2}$ in the denominator of (15) is negligible and can be ignored. Under this regime, the SI varies almost linearly with $\bar{n}$ as

18 $$\begin{array}{rcl}\delta (\bar{n}) & \approx  & \frac{2{{\rm{\Gamma }}}^{2}{\rm{\Omega }}}{4{{\rm{\Omega }}}^{2}+{\gamma }^{2}}+\frac{8{g}_{0}^{2}{\rm{\Omega }}(4{{\rm{\Omega }}}^{2}+{\gamma }^{2}+{{\rm{\Gamma }}}^{2})}{{({\gamma }^{2}+4{{\rm{\Omega }}}^{2})}^{2}}\bar{n}\\  & \approx  & \frac{2{g}_{0}^{2}}{{\rm{\Omega }}}\bar{n}.\end{array}$$

This result is also well in complete agreement with the expression obtained earlier for the SI^15^ in the limit of *g*_0_ ≪ Ω given as ${g}_{0}^{2}/{\rm{\Omega }}$, considering that a factor of $\frac{1}{2}$ must be added as a result of different definition of *δ*.

The first immediate conclusion which can be obtained from (18) is that the SI *δ* is always positive, meaning that the detuning frequency of red-sideband should be always a bit larger in magnitude than the blue-sideband. This also agrees with the previous findings of higher-order operator algebra^15^.

Based on (18), in the weakly-coupling limit of ${g}_{0}\sqrt{\bar{n}} < {\rm{\Gamma }}$, the normalized SI defined as $\bar{\delta }=\delta /{\rm{\Omega }}$ can be simply approximated as a function of mechanical frequency as $\bar{\delta }({\rm{\Omega }})\approx \frac{1}{2}{{\rm{\Gamma }}}^{2}/({{\rm{\Omega }}}^{2}+\frac{1}{4}{\gamma }^{2})\approx \frac{1}{2}{{\rm{\Gamma }}}^{2}/{{\rm{\Omega }}}^{2}$. This approximation is fairly convenient in explanation of SI in Raman scattering of various materials which exhibit multiple Raman lines, as shown comprehensively in the Supplementary Information.

### Optimum operation

The unique mathematical form of (14) which is composed of a first- and second-order polynomials in terms of $\bar{n}$ respectively, offers a clear maximum at a certain optimum intracavity photon number ${\bar{n}}_{{\rm{\max }}}$. To do this, let us first define the dimensionless constants $\alpha =4{g}_{0}^{2}/{{\rm{\Gamma }}}^{2}$ and *β* already defined under (16), *ϑ* = *γ*/2Ω, and *ψ* = Γ^2^/2Ω^2^. Then, the SI (17) can be rewritten as

19 $$\delta (\bar{n})={\rm{\Omega }}\psi \frac{1+\alpha \bar{n}}{{{\vartheta }}^{2}+{(1-\beta \bar{n})}^{2}}.$$

This offers the optimum intracavity photon number and thus the maximum attainable SI as

20 $$\begin{array}{rcl}{\bar{n}}_{{\rm{\max }}} & = & \frac{\sqrt{{(\alpha +\beta )}^{2}+{\alpha }^{2}{{\vartheta }}^{2}}}{\alpha \beta }-\frac{1}{\alpha }\approx \frac{1}{\beta }=\frac{{{\rm{\Omega }}}^{2}}{2{g}_{0}^{2}},\\ {\delta }_{{\rm{\max }}} & = & \delta ({\bar{n}}_{{\rm{\max }}})\approx \frac{4{{\rm{\Omega }}}^{3}}{{\gamma }^{2}}.\end{array}$$

We should take note of the fact that the maximum practically measureable SI, which occurs at the optimum intracavity photon number ${\bar{n}}_{{\rm{\max }}}={{\rm{\Omega }}}^{2}/2{g}_{0}^{2}$, is actually at the onset of bistability, and under practical conditions, heating due to optical losses in dielectric.

**Figure 2:**
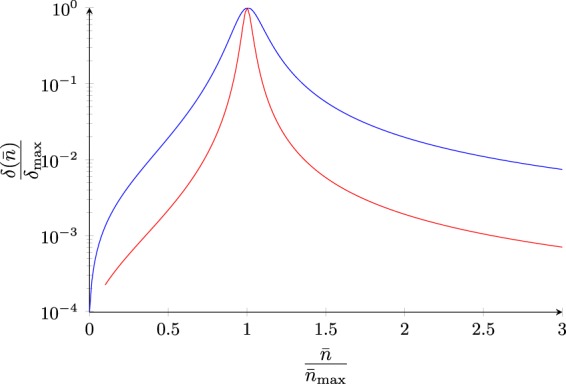
Variation of SI around the maximum point in terms of various settings of parameters: (blue) *α* = 0.1, *β* = 10^−3^, and *ϑ* = 0.1 (red) *α* = 0.1, *β* = 10^−2^, and *ϑ* = 3.16 × 10^−2^. Normalization on the vertical axis has been done to drop dependence of *δ* on Ω*ψ* in (19). The behavior versus intracavity photon number $\bar{n}$ is clearly not Lorentzian.

In Fig. 2, variation of SI versus intracavity photon number and in terms of different settings for input parameters {*α*, *β*, *ϑ*} is illustrated.

### Operation regimes

Another very important result which can be drawn from the above discussions, is marking the boundaries of linear, weakly nonlinear, and strongly nonlinear interaction regimes in quantum optomechanics. This follows by normalizing *δ* with respect to the mechanical frequency Ω first, as $\bar{\delta }=\delta /{\rm{\Omega }}$.

- Fully Linear: This regime is easily given by $\bar{n}\ll {\bar{n}}_{{\rm{\max }}}$, where intracavity photon number is essentially too low to cause any appreciable SI. Here, the behavior of normalized SI is proportional to $\bar{n}$.
- Weakly Nonlinear: This regime is next given by $\bar{n} \sim {\bar{n}}_{{\rm{\max }}}$ around the optimum operation point, where the SI rises to attain a maximum. The behavior of normalized SI is nearly Lorentzian centered at $\bar{n}={\bar{n}}_{{\rm{\max }}}$, with an intracavity photon number linewidth of ${\rm{\Delta }}\bar{n}=\vartheta {\bar{n}}_{{\rm{\max }}}$.
- Stongly Nonlinear: This regime at larger intracavity photon numbers satisfying $\bar{n}\gg {\bar{n}}_{{\rm{\max }}}$ will push the system into strongly nonlinear regime where the SI quickly start to fade away. Here, the behavior of normalized SI is inversely proportional to $\bar{n}$.

These three behaviors in above operation regimes can be respectively displayed as

21 $$\begin{array}{lll}\bar{\delta }(\bar{n}) &  \sim  & (\frac{\bar{n}}{{\bar{n}}_{{\rm{\max }}}}){\bar{\delta }}_{{\rm{\max }}},\,\,\,\bar{n}\ll {\bar{n}}_{{\rm{\max }}},\\ \bar{\delta }(\bar{n}) &  \sim  & {\{1+{{\vartheta }}^{-2}{(\frac{\bar{n}}{{\bar{n}}_{{\rm{\max }}}}-1)}^{2}\}}^{-1}{\bar{\delta }}_{{\rm{\max }}},\,\,\,\bar{n} \sim {\bar{n}}_{{\rm{\max }}},\\ \bar{\delta }(\bar{n}) &  \sim  & (\frac{{\bar{n}}_{{\rm{\max }}}}{\bar{n}}){\bar{\delta }}_{{\rm{\max }}},\,\,\,\bar{n}\gg {\bar{n}}_{{\rm{\max }}}.\end{array}$$

### Fundamental symmetries

It is easy to verify that the SI does not violate the two fundamental symmetries of the nature. Here, both the time-reversal symmetry as well as the conservation of energy are preserved. The energy of scattered red- and blue- photons $\hslash \omega \mp \hslash {\rm{\Omega }}$ is normally expected to be within the energy of one phonon *ℏ*Ω where *ω* is the angular frequency of incident electromagnetic radiation. Per every annihilated photon, exactly one phonon is either annihilated, giving rise to a blue-shifted photon, or one phonon is created, giving rise to a red-shifted photon.

However, not all phonons are having exactly the same energies. This is permissible by the non-vanishing mechanical linewidth Γ > 0 of the cavity. One should expect that once this quantity vanishes, the SI is gone, since it is by (19) proportional to Γ^2^. Hence, basically it should be not contradictory to have a possible non-zero SI.

With regard to the time-reversal symmetry, one must take notice of the fact that all optical frequencies are physically positive, since we first must move back out of the rotating reference frame. For instance, the blue- and red-scattered photons have frequencies given by ${\omega }_{b}={\omega }_{c}+{\rm{\Omega }}-\frac{1}{2}\delta$ and ${\omega }_{r}={\omega }_{c}-{\rm{\Omega }}-\frac{1}{2}\delta$. Therefore, blue and red processes are not time-reversed processes of each other, as they both stay on the positive frequency axis. Negative frequency images corresponding to both processes do however exist and exactly satisfy the time-reversal.

### Optomechanical experiments

To the best knowledge of the author, at least two very high resolution optomechanical experiments on side-band resolved samples, reveal the existence of SI, shown in Table 1. One experiment^22^ exhibits a normalized SI of (3.5 ± 0.9) × 10^−6^, equivalent to *δ* = 2*π* × (142 ± 36) H*z* with Ω = 2*π* × 40.5968 MHz. There exists a much more recent experiment, where the observed normalized SI is much stronger (9.23 ± 0.66) × 10^−4^ ^23^.

**Table 1:** Side-band inequivalence in absolute values and normalized quantities in optomechanical experiments. Both experiments are carried out on side-band resolved cavities and have sufficiently high resolution to look for deviations from ideal expected case. Further examples are analyzed and discussed in Supplementary Information.

Experiment	SI *δ*/(2*π*)	Normalized SI $\bar{{\boldsymbol{\delta }}}={\boldsymbol{\delta }}/{\boldsymbol{\Omega }}$	Remarks
Schleisser *et al*.^22^	142 ± 36 H*z*	(3.5 ± 0.9) × 10^−6^	Resonant pump^a^
Sudhir *et al*.^23^	3.97 ± 0.284 k*Hz*	(9.23 ± 0.66) × 10^−4^	Out-of-loop heterodyne spectra^b^

^a^Analysis done on Fig. 5b^22^ and the data corresponding to the side-bands at resonant Δ = 0 pumping, which are much more visible and reliable. For near-blue resonant pump with Δ ≈ −Ω deviations lie within the error margin and are thus inconclusive. Coordinates of side-band Lorentzians were taken from ultra high-resolution digitalization of the graph.

^b^Analysis done on the open-loop configuration with no feedback in Fig. 4b^23^.

Inclusion of higher-order side-bands up to the *n*− th order would have caused an algebraic equation of the order 2*n* in place of the quadratic (12), which is in general impossible to solve explicitly. Furthermore, the numerical advantage of treating such a generalized case would be only minor. With regard to the imaginary part ℑ[*δ*] which was discarded, even if it were non-zero in the exact analysis, it could have resulted for instance in modification of linewidths. This calls for a deeper study of this subject, and it is also worth mentioning that the asymmetry of linewidths in optomechanics has recently been studied elsewhere in the linearized regime^24^.

### Raman scattering

Remarkably, the Stokes and anti-Stokes peaks of Raman spectra also exhibit the same phenomenon of SI. This has so far skipped the attention since normally the nonlinear interaction rate causing the formation of Raman scattering is too small for majority of bulk materials and liquids. Again, a full linear theory of Raman scattering^25^ disallows SI. However, with the recent advent of low-dimensional materials, optical nonlinear interactions leading to Raman scattering^26,31–33,38^ and Kerr effect^34^ are available at much stronger rates.

Table 2 provides a summary of measurable SI based on the reported Raman scattering of different materials. Calculations of normalized SI and errors are done according to the best possible resolution of measurement graphs. It has to be noted that for every given material, the SI is a strong function of scattering order, excitation wavelength, polarization, angle of incidence, as well as intensity. Therefore, it is not possible to reconstruct a fit to varying function for *δ* as (19). However, the existence of a previously unnoticed SI, which is always leaning toward the red side-band seems to be conclusive. Therefore, a rigorous quantum theory of SI for Raman scattering from continuum optomechanics is yet to be developed, such as the one recently developed for materials^25^.

**Table 2:** Side-band inequivalence in Raman spectra of various materials, computed based on the highest-resolution available measurements. The last four rows under the single separating horizontal line are obtained by direct calculation on available high-resolution measurement data. Confidence interval for these last three measurements is remarkably significant, leaving no doubt in existence of SI. Further examples are analyzed and discussed in Supplementary Information.

Material	Normalized SI $\bar{{\boldsymbol{\delta }}}={\boldsymbol{\delta }}/{\boldsymbol{\Omega }}$	Remarks
MoTe_2_	(7 ± 0.8) × 10^−2^	6-layer^35^
MoS_2_	(3 ± 1) × 10^−3^	6-layer^36^
	(7.8 ± 1.5) × 10^−3^	flake^37^
CNT^a^	(6.0 ± 2.1) × 10^−3^	(10, 5) SC^b^^27^
	(2 ± 1) × 10^−3^	1st-order; SC^b^^28^
	(1.22 ± 0.13) × 10^−2^	2nd-order; SC^b^^28^
	(1.5 ± 0.18) × 10^−2^	single-wall^29^
	(2.11 ± 0.35) × 10^−2^	single-wall^30^
Gr^c^	(8.0 ± 1.6) × 10^−3^	4-layer^39^
	(5 ± 1.7) × 10^−3^	3-layer^39^
	(1.63 ± 0.48) × 10^−2^	(1, 2)-twisted^40^
	(2.2 ± 1.09) × 10^−2^	C_31_ (1, 3)-twisted^d^^40^
	(3.6 ± 1.8) × 10^−2^	C_32_ (1, 3)-twisted^d^^40^
C	(1.15 ± 0.57) × 10^−2^	Bulk^41^
S^e^	(1.03 ± 0.25) × 10^−2^	1st-order; grains^37^
	(7.2 ± 1.8) × 10^−3^	2nd-order; grains^37^
Ethanol	(1.8 ± 0.4)×10^−3^	1st-order; EX^f^^42^
H_2_	(4.7 ± 0.223) × 10^−4^	MOM^g^^43^
Gr	(2.67 ± 0.074) × 10^−3^	*P*op = 7.0 mW^h^^44^
	(1.67 ± 0.165) × 10^−3^	*P*op = 3.8 mW^h^^44^
	(0.67 ± 0.603) × 10^−3^	*P*op = 1.8 mW^h^^44^
B4^i^	(3.4 j ± 0.18) × 10^−3^	1090 nm line^45,46^
SRA^k^	(1.424 ± 0.01) × 10^−3^	Yb-doped fiber^47,48^

^a^CNT: Carbon Nano-tube.

^b^SC: Semiconducting.

^c^Gr: Graphene.

^d^1.96 eV excitation.

^e^S: Sulphur.

^f^EX: Excitation at 532 nm.

^g^MOM: Molecular Optical Modulation; Measurement at 1015 kPa done on ortho −H_2_, which has a 17.6THz rotational motion frequency. Experimental data received from the authors through private communication.

^h^3ps Ultrafast Excitation; Experimental data received from the authors through private communication. Data has to be shifted to adjust for *δ*(0) = 0, and exhibits a linear rate of 3.867 × 10^−4^/mW versus input power, in agreement with the weakly nonlinear regime of (21) as $\delta (\bar{n})\propto \bar{n}$.

^i^B4: Single Biphenyl-4-thiol molecule in monolayer confined to optical picocavity; Experimental data available online^46^.

^j^4-point moving average applied. Guassian 10-point filtering results in the much larger value of 1.06 × 10^−2^.

^k^SRA: Stimulated Raman Amplifier; Experimental data available online^48^.

In the end and following the above, let us now plug-in (4) and (7) into the first of (10). Some simplifications, while ignoring the inequilibrium quantum thermal effects on the population of side-bands, gives the equation

22 $${\bar{n}}_{r}-{\bar{n}}_{b}\approx (\frac{{\rm{\Omega }}\bar{n}}{{{\rm{\Omega }}}^{2}+\frac{1}{4}{{\rm{\Gamma }}}^{2}})\delta \approx \frac{\bar{n}}{{\rm{\Omega }}}\delta .$$

Here, ${\bar{n}}_{r}=|{\bar{a}}_{r}{|}^{2}$ and ${\bar{n}}_{b}=|{\bar{a}}_{b}{|}^{2}$ respectively refer to the number of scattered photons unto red and blue side-bands. Then, from (19), and assuming that $\bar{N}=({\bar{n}}_{r}-{\bar{n}}_{b})/\bar{n}$ denotes the normalized asymmetry of side-bands, we get

23 $$\bar{N}(\bar{n})=\frac{\psi (1+\alpha \bar{n})}{{{\vartheta }}^{2}+{(1-\beta \bar{n})}^{2}}.$$

Accordingly the asymmetry is increases up to a positive maximum, before decreasing back to zero at sufficiently high powers.

### Side-band inequivalence in other areas

Finally, it is easy to see that the same nonlinear symmetry breaking can lead to asymmetry in the particle pair production or parametric down conversion, which can be considered as the dual of optomechanical process^49,50^. In order to observe this fact, consider an optomechanical system with a mechanical frequency roughly double the optical frequency Ω ≈ 2*ω*. If the mechanics is driven strong enough at the frequnecy Ω, then the effective interaction Hamiltonian will be simply ${{\mathbb{H}}}_{{\rm{eff}}}=i\hslash g({\hat{a}}^{\dagger }{\hat{a}}^{\dagger }\hat{b}-\hat{a}\hat{a}{\hat{b}}^{\dagger })$, where a phonon with energy *ℏ*Ω dissociates into two photons with energies *ℏ*(*ω* ± *δ*) with *δ* representing the corresponding symmetry breaking in pair frequencies caused by SI. Parametric down conversion for phonons has recently been observed and reported, too^51^.

Also, based on the duality of effective interaction Hamiltonian in linear electro-optic modulation (within the validity of rotating wave approximation), with the optomechanical Hamiltonian^52–54^, one could predict that the same nonlinear inequivalence to appear in relevant experiments, too. As an example, optical modulation of Hydrogen^43^ is already shown in Table 2 to exhibit this phenomenon. Hence, further implications could be expected in communications technology and filtering, where precise positioning of side-bands are of importance. Similar arguments should be valid for enhanced Raman scattering of single molecules by localized plasmonic resonances as well^55^.

## Conclusions

We presented a complete analysis of SI in quantum optomechanics, and showed it undergoes a maximum and obtained closed-form expressions for optimum intracavity photon number as well as maximum attainable SI. We classified the operation into the linear, weakly nonlinear, and strongly nonlinear regimes, in which the behavior of system is markedly different. The results of this investigation can provide the accuracy constraints as well as necessary experimental set up to resolve the elusive SI. Analysis of high resolution measurements of Raman scattering for different materials confirms the existence of SI. One could speculate that precise measurement of the variation of side-band inequialence in terms of various system parameters could provide further insight into unexplored nonlinear properties of different material.

## Supplementary information

Further Evidence from Published Literature
