# Supplementary material for: Analysis of Side-band Inequivalence
Source: Sci Rep. 2019 Jun 24;9:9075. doi: 10.1038/s41598-019-45580-7 (PMC6591420; doi:10.1038/s41598-019-45580-7)
Supplement: Supplementary file 1 — Further Evidence from Published Literature [file 41598_2019_45580_MOESM1_ESM.pdf]

Supplementary Information: Further Evidence from Published Literature

Analysis of Side-band Inequivalence

Sina Khorasani

"Plus un fait est extraordinaire, plus il a besoin d'être appuyé de fortes preuves; car, ceux qui l'attestent pouvant ou tromper ou avoir été trompés, ces deux causes sont d'autant plus probables que la réalité du fait l'est moins en elle-même."
Pierre-Simon Laplace [S1]

Abstract

This document includes a comprehensive and categorized referenced listing of published experiments which exhibit detectable Side-band Inequivalence (SI). Because of the resonant behavior of SI as found for δ in (14) of the main article, most experiments actually appear to have missed that resonance. Only those which hit the near-resonance criteria could have resulted in noticeable effect. Traces of SI could be found in a wide class of physically equivalent experimental platforms including Quantum Optomechanics, Ion and Paul Traps, Electrooptic Modulation, Brillouin Scattering, and Raman scattering. In what follows, we present an reasonably convincing overview of published available works, and divide them into various categories, and also discuss the existence of SI in approximate analytical solutions of classical optomechanics.

Disclaimer

Contents of this document reflect the opinion of the author of present study (S.K.), and not necessarily those of original authors and/or laboratories, where respective works were carried out and here are cited. In some cases, the original authors were contacted and few agreed to the ultimate conclusions on SI drawn here while most remained unjudgemental. It is to be understood that referenced works contain public and extremely valuable information, and every effort has been made to ensure that the accuracy of analyses be kept as high as possible. Wherever raw measurement data has been available publicly or been graciously shared by the experimenters, calculations are carried out directly on numerical data. Otherwise, high-resolution digitization of graphs has been used to derive side-band inequivalences and frequencies to the best possible accuracy.

S1 Quantum Optomechanics

The family of optomechanical interactions having the form $\mathbb{H}_i = \hbar g_0 \hat{a}^\dagger \hat{a}(\hat{b} + \hat{b}^\dagger)$ could be found in cavity quantum optomechanics, cavity quantum electromechanics, and quantum magnonics [S2–S5]. In all these experiments, interactions take place inside a doubly-confined cavity where both photons and phonons are kept interacting subject to decay. This platform provides the most straightforward and reliable source of SI as long as it is large enough to be measured. Typically, it is too small here and for all practical reasons, normalized SI in cavity quantum optomechanics is normally in the range of 10^{-6} to 10^{-4}. This calls for very precise fabrication, calibration and stable measurements, which makes this type the most difficult way to obtain SI.

In cavity quantum optomechanics, there are three recognized and analyzed types of asymmetry between red and blue side-bands:

1. Side-band Asymmetry: which is a quantum effect connected to different thermal phonon occupation of side-bands [S2]. Under thermal equilibrium the red side-band is more populated since it has lower frequency.
2. Line-width Asymmetry: which is due to the different linewidths of red and blue side-bands. This phenomenon is also recently analyzed and now well understood [S7].
3. Side-band Inequivalence (SI): which is the topic of this article under consideration.

Optomechanical experiments may exhibit a tiny yet detectable SI [S8–S10], which is always biased towards red. A remarkable optomechanical experiment [S9] on an optomechanical crystal reports precise measurement of side-bands at various pump levels without and with cooling tone. In absence of cooling tone, the location of side-bands asymmetrically moves almost linearly with the measured photon population, up to 4kHz [S9]. Another very high precision experiment [S8] on micro-toroids, exhibits markedly frequency asymmetry both in transmission and power spectral density of side-bands. The difference in particular becomes quite noticeable for the second-order side-bands, which do not quite fit well to the linearized model. The SI here is around 142Hz.

There exist another report in solid-state cavity quantum optomechanics with sufficiently high resolution [S10] and right conditions which exhibits SI, too. Under thermal equilibrium and absence of cooling tone, the side-bands are well resolved and sharp enough to detect frequency differences, and again here SI is biased towards red. Analysis of a report on motional side-bands in solid-state optomechanical cooling by controlling the interaction with surface acoustic waves [S11] in a similar manner shows a noticeable SI $\bar{\delta} \approx 0.74\%$, which is of course biased towards red.

It should be noted that optically confined particles in side-band resolved regime can display remarkably strong SI. Given the fact that the nature of these experiments are markedly different than cavity quantum optomechanics, it is quite exciting indeed to observe the fact that SI does survive. A recent such experiment [S12], with the reported uncertainties taken into account, exhibits an SI as large as $\bar{\delta} = 0.8\%$, if we assume that the zero calibration is sufficiently more accurate than 0.1 kHz. Otherwise, referencing with respect to the fitted central resonance gives $\bar{\delta} = 0.43\% \pm 0.18\%$. In any case, it is positive as expected within a reasonable confidence interval, that is the red peak is definitely further away from the resonance as opposed to the blue. For this experiment, the raw measurement data was made available to the author, providing an accurate analaysis, as depicted in Fig. S1. Optical confinement of a single-atom using tweezers [S13] also leads to measurement of single-atom sideband spectra,

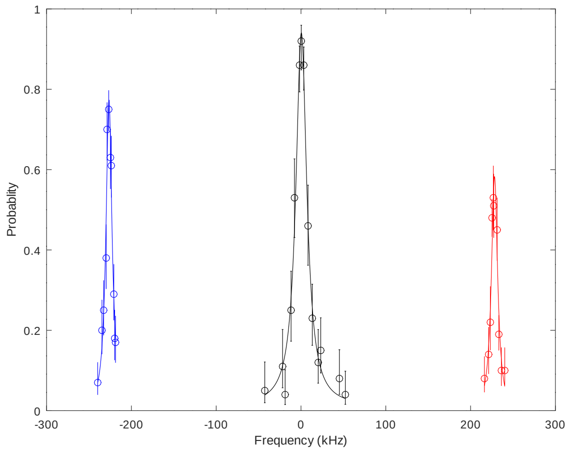

Figure S1. Motional sidebands of an optically trapped ion in a tweezer before sideband cooling obtained from spectroscopic raw measurement data [S12].

which in absence of cooling can be measured to have a normalized SI of $\bar{\delta} = 1.04\%$.

In a very recent landmark experiment carried out [S14] side-bands were generated as a result of controlled detuned acoustooptic modulation, where detuning frequency effectively replaces modulation frequency. The authors have made the measurement data available online [S15] from which the SI may be calculated with respect to the modulation frequency. The behavior of SI here is dependent on the parameters of modulator and not the optomechanical device, so we had to look for fitting parameters. The appropriate expression in the limit of very small Γ follows (17) in the main article. Assuming $\gamma \approx \kappa$ and $g = g_0\sqrt{\bar{n}}$, it takes the form

$$\bar{\delta}(\Omega) = \frac{8g^2}{\kappa^2}\frac{\Omega^2 \text{sgn}[\Omega]}{\Omega^2 + 4\frac{1}{\kappa^2}(\Omega^2 - 2g^2)^2}, \tag{S1}$$

noticing that Ω represents the detuning frequency and can be positive or negative. Hence, the normalized SI $\bar{\delta}$ may be considered as a function of Ω, too, and $\text{sgn}(\cdot)$ is the sign function. This expression (S1) very well fits the calculated behavior based on the analysis of raw measurement data [S15], giving a remarkably nice fit. However, it has to be pointed out again that the optimum fitting parameters $(\kappa, g) = 2\pi(620, 90)$kHz are irrelevant to the optomechanical system parameters.

Breathing Solutions

In the fully classical approximation, the nonlinear optomechanical equations read

$$\begin{aligned} \frac{d}{dt}\Upsilon &= \left(i\Delta - \frac{1}{2}\kappa\right)\Upsilon + ig_0\Upsilon(\Phi + \Phi^*), \\ \frac{d}{dt}\Phi &= \left(-i\Omega - \frac{1}{2}\Gamma\right)\Phi + ig_0\Upsilon^*\Upsilon. \end{aligned} \tag{S2}$$

Under the assumption of slowly varying amplitude $|d\Upsilon/dt| << \Omega|\Upsilon|$ and large optical quality factor $\kappa << |\Delta|$, the set of equations (S2) admits an analytical solution for the optical field of the form

$$\Upsilon(t) = \frac{\theta e^{i\theta^2\tau}}{\sqrt{2D/|\Delta|}}\left[\frac{2\iota^2\cosh(\vartheta\tau) - i2\iota\vartheta\sinh(\vartheta\tau)}{\cosh(\vartheta\tau) - \sqrt{1-\iota^2}} - 1\right], \tag{S3}$$

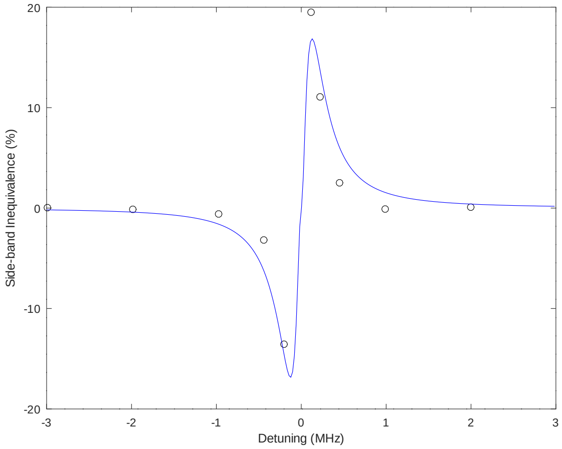

Figure S2. Calculated SI from raw measurement data [S15] and fitting function based on (S1) with modulator parameters $g = 2\pi \times 90\text{kHz}$ and $\kappa = 2\pi \times 620\text{kHz}$.

referred to Akhmediev breathers [S16, S17]. Here, $\tau = |\Delta|t$ is normalized time, θ is a real constant to be determined, D is defined in (15), and $\vartheta = -2\theta^2\imath\sqrt{1-\imath^2}$. For $\imath < 1$ solutions are breathing soliton-like, while for $\imath > 1$ solutions become nonlinear oscillatory waves.

When $\imath < 1$, then the expression within the brackets is composed of an even real part and an odd imaginary part. Hence, the Fourier transform of this hyperbolic expression within the brackets is real-valued, which we here denoted by $\mathscr{G}(w)$. Obviously, the magnitude of Fourier transform, or the spectrum $|\mathscr{G}(w)|$ no longer needs to be even. Hence, the Fourier transform of $\Upsilon(t)$, which is given by $\mathscr{J}(w) = \mathscr{F}\{\Upsilon(t)\}(w)$ is simply the shifted transform $\mathscr{G}(w-\theta^2|\Delta|)$, and the amount of frequency shift is nothing but the SI. This fact is a direct result of the multiplying term $\exp(i\theta^2\tau)$ in the analytical solution (S3), and thus the SI.

While, the algebraic hyperbolic form of (S3) with $\imath < 1$ disallows analytical evaluation of the spectrum $\mathscr{J}(w)$, however, calculation of $\mathscr{J}(w)$ from (S3) with $\imath > 1$, which leads to periodic nonlinear oscillatory solutions, becomes explicitly possible as shown in the following. To do so, we may proceed with the substitution $\imath = |(\Omega + i\Gamma/2)/g| \approx \Omega/g$ where $g = g_0\sqrt{n}$ is the enhanced optomechanical interaction rate, which in the weakly-coupled limit obviously satisfies $\imath >> 1$. Hence, we have $i\vartheta = 2\theta^2\imath\sqrt{\imath^2-1} \approx 2\theta^2\imath^2$. Now, we can rearrange (S3) as

$$\Upsilon(t) = \frac{2\imath\theta e^{i\theta^2\tau}}{\sqrt{2D|\Delta|}}\left[\frac{\imath|\Delta|\cos(\varpi t) + i\varpi\sin(\varpi t)}{\cos(\varpi t) - i\sqrt{\imath^2-1}} - \frac{|\Delta|}{2\imath}\right], \tag{S4}$$

where the replacement $i\vartheta\tau = \varpi t$ has been made and $\varpi = \Omega + \delta\Omega$ is the shifted mechanical frequency while taking the optical spring effect $\delta\Omega$ into account. The expression within brackets in (S4) can be simplified using trigonometric identities as

$$\begin{aligned}\Upsilon(t) &= \Xi e^{i\frac{1}{2}\delta t}\left[\frac{(\imath|\Delta|+\varpi)e^{i\varpi t} + (\imath|\Delta|-\varpi)e^{-i\varpi t}}{e^{i\varpi t}+e^{-i\varpi t}-2i\sqrt{\imath^2-1}} - \frac{|\Delta|}{2\imath}\right] \qquad \text{(S5)}\\ &= \Xi e^{i\frac{1}{2}\delta t}\left[\frac{2i\sqrt{\imath^2-1}(\imath|\Delta|+\varpi) - 2\varpi e^{-i\varpi t}}{e^{i\varpi t}+e^{-i\varpi t}-2i\sqrt{\imath^2-1}} + \frac{2\imath(\imath|\Delta|+\varpi)-|\Delta|}{2\imath}\right]\\ &= 2\Xi e^{i\frac{1}{2}\delta t}\left[\frac{i\varsigma - \varpi e^{-i\varpi t}}{e^{i\varpi t}+e^{-i\varpi t}-2i\sqrt{\imath^2-1}}\right] + \Xi\left[\frac{2\imath(\imath|\Delta|+\varpi)-|\Delta|}{2\imath}\right]e^{i\frac{1}{2}\delta t}\\ &= i\Psi e^{\frac{1}{2}\delta t}f(t) + \Lambda e^{i\frac{1}{2}\delta t},\end{aligned}$$

in which $\Xi = \sqrt{2}\imath\theta/\sqrt{D}$, $\Lambda = \Xi(2|\Delta|\imath^2 - 2\varpi\imath - |\Delta|)/2\imath$, $\varsigma = \sqrt{\imath^2-1}(\imath|\Delta|+\varpi)$, and $\Psi = 2\Xi\varsigma$. We here may notice that $\delta = \theta^2|\Delta| > 0$ is nothing but the SI. The function $f(t)$ can be rewritten as

$$\begin{aligned}f(t) &= \frac{1+i\frac{\varpi}{\varsigma}e^{-i\varpi t}}{e^{i\varpi t}+e^{-i\varpi t}-2i\sqrt{\imath^2-1}} = \frac{e^{i\varpi t}+i\varkappa}{(e^{i\varpi t}-i\sqrt{\imath^2-1})^2+\imath^2} \qquad \text{(S6)}\\ &= \frac{e^{i\varpi t}+i\varkappa}{(e^{i\varpi t}-i\zeta^+)(e^{i\varpi t}-i\zeta^-)} = \frac{1}{2\imath}\frac{\zeta^+ + \varkappa}{e^{i\varpi t}-i\zeta^+} - \frac{1}{2\imath}\frac{\zeta^- + \varkappa}{e^{i\varpi t}-i\zeta^-}\\ &= h(e^{i\varpi t}),\end{aligned}$$

where the replacement $\zeta^{\pm} = \pm\iota + \sqrt{\iota^2 - 1}$ has been made and $\varkappa = \varpi/\varsigma$ with $\zeta^+\zeta^- = -1$, $\zeta^+ > 0$ and $\zeta^- < 0$ respectively lying in the upper and lower half-complex planes. Obviously, the pole at $i\zeta^+$ falls outside the unit circle, and only the pole at $i\zeta^-$ remains inside the unit circle. Hence, $\varkappa << -\zeta^- << 1 << \zeta^+$. These will be needed later to derive the spectrum of (S4).

All remains now is to solve $i\vartheta\tau = \varpi t$, which gives rise to the approximate solution

$$\delta \approx \frac{2\varpi}{\iota\sqrt{\iota^2-1}} = 2g^2 \frac{\Omega + \delta\Omega}{\sqrt{\Omega^2 + \frac{1}{4}\Gamma^2}\sqrt{\Omega^2 + \frac{1}{4}\Gamma^2 - g^2}}. \tag{S7}$$

This can be further written as

$$\frac{\delta}{2} \approx \frac{\Omega + \delta\Omega}{\iota^2} = g^2 \frac{\Omega + \delta\Omega}{\Omega^2 + \frac{1}{4}\Gamma^2} \approx \frac{g^2}{\Omega} + \frac{g^2}{\Omega^2}\delta\Omega \approx \frac{g^2}{\Omega} - \frac{2g^4\Delta}{\Omega^2}\frac{(\Omega^2 - \frac{1}{4}\kappa^2)}{(\Omega^2 + \frac{1}{4}\kappa^2)^2}, \tag{S8}$$

since $\delta\Omega \approx -2g^2\Delta(\Omega^2 - \frac{1}{4}\kappa^2)/(\Omega^2 + \frac{1}{4}\kappa^2)^2$. It is here seen that the correction arising from spring effect is $O(g^4)$ while the SI is $O(g^2)$. Hence, the normalized SI $\bar{\delta} = \delta/\Omega$ can be finally approximated for the side-band resolved regime $\Omega >> \kappa$ as

$$\bar{\delta} \approx \frac{2g^2}{\Omega^2}\left[1 - \frac{2g^2\Delta(\Omega^2 - \frac{1}{4}\kappa^2)}{\Omega(\Omega^2 + \frac{1}{4}\kappa^2)^2}\right] \approx \frac{2g^2}{\Omega^2}\left[1 - \frac{2g^2}{\Omega^3}\Delta\right], \tag{S9}$$

showing that the correction of optomechanical spring effect to the ansatz (1), ultimately yielding the expression for SI (14) had been safely ignored indeed. There is also a higher-order correction to the optomechanical spring effect $\delta\Omega$ [S2] as a result of non-zero coherent phonon population $\bar{m}$, which results in an extra correction to (S8) by replacing $g = g_0\sqrt{\bar{n}}$ within the brackets of (S9) with $g \approx g_0\sqrt{\bar{n} + \bar{m} + 1}$. But this leaves our derivations and conclusions regarding the SI unchanged.

This solution (S5) is a bi-periodic and complex-valued product of two periodic functions $f(t)$ and $\exp(i\delta t)$. Fourier transform of the periodic function $f(t) = f(t + 2\pi/\varpi)$ defined as $F(w) = \frac{1}{2\pi}\int_{-\infty}^{\infty} f(t)e^{-iwt}dt$ is starightforward to obtain. In fact, one should have $F(w) = \sum_\nu f_\nu \delta(w - \nu\varpi)$, where $\delta(\cdot)$ are Dirac's delta functions, and $f_0 = f(1)$ while $f_\nu = \exp(i2\pi/\nu), \nu \neq 0$. Hence, it is straightforward to see that how its spectrum looks like. Defining the spectrum of $f(t)$ as $|F(w)|$ in the Fourier domain w simply is $F(w) = \sum_\nu |f_\nu|\delta(w - \nu\varpi)$, which consists of Dirac deltas at $w = \pm\nu\varpi \approx \pm\nu\Omega, \nu \in \mathbb{N}$ corresponding to the side-bands. A practical system obviously does not exactly follow the breather solution (S3) and hence side-bands all have finite non-zero linewidths. Ultimately, we have $I(w) = |\mathscr{J}(w)|$.

Therefore the ultimate spectrum of the cavity within the approximation of breather solutions is $I(w) \approx |F(w - \frac{1}{2}\delta)| + R(w)$, where $R(w)$ is the reflection from cavity at central resonance $w = 0$. Since the reflected central resonance $R(w)$ normally masks out the zeroth harmonic, therefore the side-bands appear to be positioned asymmetrically in frequency equal to the SI δ. Hence, $I(w)$ may be written conveniently as

$$\begin{aligned}
I(w) &= |\tfrac{1}{2\pi}\Lambda + i\Psi f_0|\delta(w - \tfrac{1}{2}\delta) + \Psi\sum_{\nu=1}^{\infty}\left[|f_\nu|\delta(w - \tfrac{1}{2}\delta - \nu\varpi) + |f_{-\nu}|\delta(w - \tfrac{1}{2}\delta + \nu\varpi)\right], \qquad \text{(S10)}\\
f_\nu &= \frac{1}{2i\varpi\pi}\oint \frac{h(z)}{z^{\nu+1}}dz,\\
&= -\left[\frac{\zeta^- + \varkappa}{2\varpi\iota(i\zeta^-)^{\nu+1}}\right] + \frac{1}{\varpi\nu!}\left[\frac{d^\nu}{dz^\nu}h(z)\right]_{z=0} u(\nu)\\
&= \left[\frac{i(\zeta^- + \varkappa)}{2\varpi\iota i^\nu(\zeta^-)^{\nu+1}}\right] - \frac{\zeta^- + \varkappa}{2\iota\varpi\nu!}\left[\frac{(-1)^\nu\nu!}{i^{\nu+1}(\zeta^-)^{\nu+1}}\right]_{z=0} u(\nu)\\
&= \left[\frac{i(\zeta^- + \varkappa)}{2\varpi\iota i^\nu(\zeta^-)^{\nu+1}}\right][1 + (-1)^\nu u(\nu)].
\end{aligned}$$

where the change of variables $z = \exp(i\varpi t)$ has taken place, and the integration is taken counter-clockwise on the unit circle in the complex $u-$plane. The only contributing pole of $h(z)$ is at $z = i\zeta^- \approx -i/2\iota^2$. Furthermore, $u(\cdot)$ is the unit-step function, which allows the second term to contribute only if $\nu \geq 0$. The function $h(z) = (z - \varkappa)/(z - i\zeta^+)(z - i\zeta^-)$ was also defined in (S6). In (S10), the positive odd harmonics identically vanish, and f_ν and $f_{-\nu}$ respectively correspond to Stokes and anti-Stokes amplitudes. There are no odd-ordered Stokes components in the breather nonlinear oscillatory wave (S4), and also anti-Stokes components diminish in strength with their order $-\nu$ increasing as $(\zeta^-)^{-\nu}$ according to (S10).

The total power $P = \int_{-\infty}^{\infty} I(w)dw$ is now simply $P \approx \sum_\nu |f_\nu|$, and total harmonic distortion shall be given by the simple expression $\text{THD} \approx \sum_{|\nu|\geq 2}|f_\nu| / \sum_{|\nu|\geq 1}|f_\nu|$. The first-order mechanical side-bands correspond to $f_{\pm 1}$ with sharp peaks located at $\pm\varpi - \frac{1}{2}\delta \approx \pm\Omega - \frac{1}{2}\delta$, confirming the initial ansatz (1) and speculation regarding the existence of SI. In summary, the breathing

analytical solution (S3) actually highlights the existence of a non-zero SI, simply because of the multiplying term $\exp(i\theta^2\tau)$ and its bi-periodic form, and furthermore SI has to be always towards red (Stokes) simply because $\theta^2 > 0$ is always positive.

The ratios of coefficients f_ν in (S10) can be estimated using binomial expansion of denominator in (S5) and the original form, resulting in approximate expressions for the ratios of side-band powers. For instance, the ratio of optical amplitude in the first-order side-bands with respect to the central resonance is roughly

$$\begin{aligned}\frac{I_1}{I_0} &= \frac{|f_1|+|f_{-1}|}{2\left|\frac{1}{2\pi}\Lambda+i\Psi f_0\right|}=\frac{\pi}{\varpi\iota}\frac{\zeta^-+\varkappa}{[\Lambda-(\zeta^-+\varkappa)\pi\Psi/(\varpi\iota\zeta^-)]} \\ &\approx \frac{\pi\zeta^-}{\varpi\iota\Lambda-\pi\Psi}.\end{aligned} \tag{S11}$$

The accuracy of breathing solutions for second- and higher-order harmonics is insufficient to obtain a meaningful ratio such as (S11), nevertheless, it exhibits a positive and unmistakable SI towards red.

S2 Ion/Paul Traps

The volume of existing literature on atomic and ion traps is truly vast, and we limit the study to a collection of selected works in this area. SI numbers are typically large and quite noticeable.

Detection of motional side-bands around 5GHz in a linear Paul trap placed on an optical cavity [S18] gives rise to $\bar{\delta} = 1.32\% \pm 0.32\%$ at anti-node and $\bar{\delta} = 1.42\% \pm 0.24\%$ at node. Another measurement [S19] clearly shows red and blue side-bands separately from which one may obtain the fairly accurate estimation of $\bar{\delta} = 0.021\% \pm 0.0021\%$. Doppler cooling on a microchip multi-segmented ion trap [S20] gives $\bar{\delta} = 0.047\% \pm 0.0078\%$. Doublet features of side-bands are very much visible in the next research [S21], which was the primary assumption as displayed in (1) at the beginning of our analysis. One report considers a high-resolution measurement of motional side-bands of trapped Ca^+ ions [S22], which contains four very sharp resonances on either side. All of these resonances exhibit significant SI and the values are $\bar{\delta} = \{1.8\% \pm 0.29\%, 1.3\% \pm 0.21\%, 0.50\% \pm 0.12\%, 0.87 \pm 0.11\%\%\}$ sorted in terms of increasing shift frequencies, shown in Fig. S3. Planar and vertical modes in ion traps can also individually have SI, as the recent measurements [S23] may give the respective values $\bar{\delta} = 0.096\%, 0.18\%$ within $\pm 0.008\%$. Axial motional sidebands in another microfabricated ion trap design [S24] are measured from which $\bar{\delta} = 0.037\% \pm 0.0085\%$ can be computed.

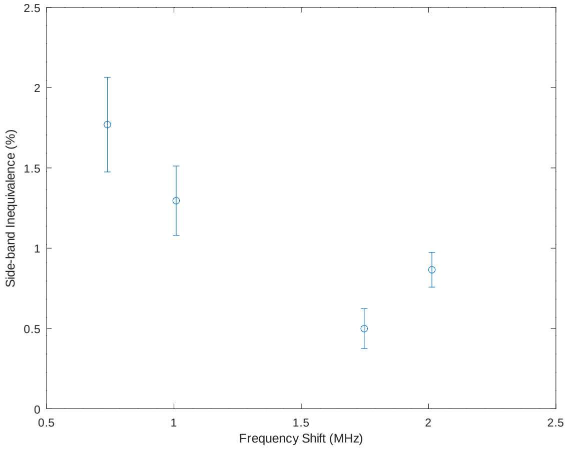

Figure S3. Side-band Inequivalences computed from digitization of microfabricated ion trap experiment [S22].

A very large SI also can be noticed in the side-bands of a calcium ion confined in a linear Paul trap [S25] as $\bar{\delta} = 6.3\% \pm 0.18\%$. Doppler cooling does not seem to remove this effect, as the value $\bar{\delta} = 0.10\% \pm 0.0070\%$ corresponds to cooled vibrational states of trapped atomic ion [S26]. Motional side-bands of two $_{40}Ca^+$ ions before sympathetic ground-state cooling [S27] show a clear SI as large as $\bar{\delta} = 0.35\% \pm 0.0030\%$. Also, an article [S28] which reports cooling of trapped $_{111}Cd^+$ ions shows $\bar{\delta} = 0.21\% \pm 0.0086\%$.

Finally, a very recent experimental study at Max-Planck Institute [S29] reports resolved-sideband cooling of an optical lattice with zoomed-out side-bands both before and after cooling takes place. The SI is so large that can be easily seen on the frequency scales equal to $\bar{\delta} = 1.9\%$. It turns out that the authors had noticed this large difference and had tried to explain it using anharmonicity of the confining potential [S30] in weak limit. It has to be mentioned that if the cause of side-band

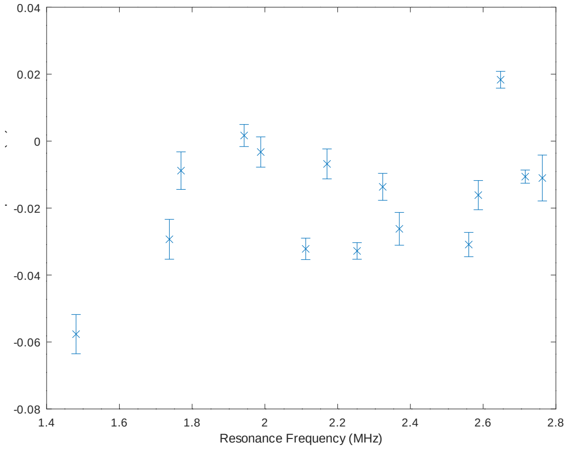

Figure S4. Side-band Inequivalences of an ion trap measured for the radial mode spectrum after Doppler cooling, computed from raw measurement data [S31].

inequivalence were anharmonicity, then the sign of expression for SI must follow the sign of anharmonic potential, that is $\delta \propto g_0$. However, the analysis in the current study shows that $\delta \propto g_0^2$ holds to a good accuracy, regardless of the sign of anharmonic optomechanical interaction.

Another article, the measured radial mode spectrum of a nine-ion linear crystal has been reported [S31] and the raw measurement data were made available to the author. There are multiple resonances on either of the blue and red sides, which are measured both after Doppler cooling and after Doppler/EIT cooling. After EIT cooling, the red side-bands almost disappear, and it has been possible to find the SI only after Doppler cooling, shown here in Fig. S4. The behavior of SI, while clearly non-zero, does not conform to the expectation and that could possibly because of Doppler cooling which dislocates resonances a bit. Nevertheless, nearly all of the 15 resonances contain non-zero SI.

Shown in Fig. S5, the SI of the motional state of a trapped $_{171}\text{Yb}^+$ ion placed in a state-dependent potential generated by a running optical lattice is computed from raw measurement data, made available to the author [S32]. Measurements here correspond to the absence of cooling of any type. Every point is furthermore the average of 100 traces, and that provides an accurate estimation of SI. The maximum normalized SI is about $\bar{\delta} = 0.99$.

Interestingly, in the limit of small optomechanical interaction rate g_0 and low optical power, one may use (18) to obtain the approximate proportionality dependence

$$\bar{\delta}(\Omega) \propto \frac{1}{\Omega^2 + \frac{1}{4}\gamma^2}, \tag{S12}$$

where Ω and γ are respectively the mechanical/modulation frequency and optomechanical decay rate. This information can be used now to check whether SI decreases with frequency in accordance to (S12). This is shown in red in Fig. S5.

As the last remark of this section, not all reported measurements apparently show SI in the desired way [S33, S34]. What these works have in common is that they include measured side-bands before (and after) cooling, and while the measureable SI seems significant, however, it is in the opposite direction. Normally, it is convenient to see the central resonance to make sure zero-referencing is accurately done, and it has come to the attention of the author that in most cases and in particular for Raman and Brillouin scattering measurements, the accurate SI cannot be found without such referencing. Anyhow, these three articles remain as unanswered questions, which need further investigation.

S3 Electrooptic Modulation

One of the pioneering works on optical fibers [S35, S36] reports the optical intensity spectra scanned with a Fabry–Perot analyzer of a single frequency laser modulated by an electrooptic modulator. It is straightforward to identify an SI as large as $0.85\% \pm 0.2\%$, due to a modulation frequency f_m on a carrier with frequency ν_0 therein.

Similarly, one may find an SI as large as $2.20\% \pm 0.27\%$ and $0.42\% \pm 0.14\%$ for the depolarized spectrum of an $LiNbO_3$ cell [S37] held at 45° and modulated at respectively at $f_1 = 3\text{GHz}$ and $f_1 = 8\text{GHz}$.

It can be easily verified here that whether (S12) holds, as the normalized SI should roughly decrease with the second power of modulation frequency (or mechanical frequency where relevant). This actually happens to be the case since one

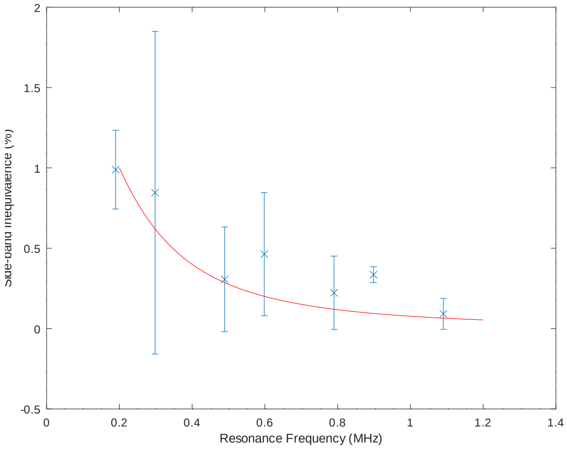

Figure S5. Side-band Inequivalences of an ion trap measured for the radial mode spectrum after Doppler cooling, computed from raw measurement data [S32]. The red fit is according to (S12) with $\gamma = 2\pi \times 63.6$kHz.

would expect then that $\bar{\delta}(f_1)/\bar{\delta}(f_2) \approx (f_2/f_1)^2$ hold. Checking the either side while taking care of error bounds gives $\bar{\delta}(f_1)/\bar{\delta}(f_2) = 0.2 \pm 0.09$ which contains $(f_2/f_1)^2 = 0.14$. These numbers are obtained from graph digitization, while such an agreement is later observed to a much higher accuracy for the case of Raman scattering where direct raw measurement data has been available.

The next reference [S38] is a thesis reporting the spectra of the Stokes/anti-Stokes signals at the output of a single-mode fiber which has been 50km long, modulated at 10.94GHz using an optical QPSK (Quadrature Phase-Shift Keying) modulator. The first three Stokes and anti-Stokes resonances are sharply measured and clearly visible, which is consistent with an unnormalized and positive SI of $\delta = (250 \pm 25)$MHz.

A recent article [S39] reports measurements of the optical spectrum of a phase modulated signal and the measurement data were made accessible to the author. The modulation provided up to seven side-bands on either side, which made the evaluation of SI possible. One would expect, likewise, that the largest SI would go to the lowest order, and that happens to be the case where an SI as large as 1.94% has been observed, which is shown in Fig. S6. There is yet another high resolution experiment on electrooptic modulation of light at telecommunications wavelength at 1545.91nm using a $LiNbO_3$ modulator with various modulation frequencies, where raw measurement data were made available [S40]. Shown in Fig. S7, the largest SI happens to occur for the lowest modulation frequency, and is very large up to 9.4%, indeed. Pressurized hydrogen as a result of molecular optical modulation [S41] has been shown to generate sidebands, due to ultrafast variation of molecule polarizability arising from coherent molecular motion. The author was also given access to the raw measurement data which was noticed to give rise to an SI of $\bar{\delta} = 0.047\%$.

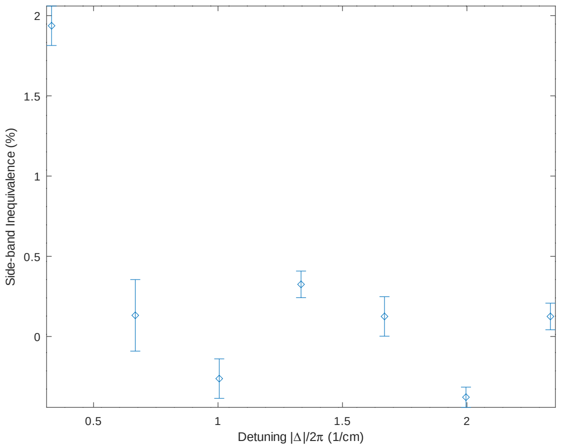

Figure S6. Side-band Inequivalence of an electrooptically modulated light computed from raw measurement data [S39].

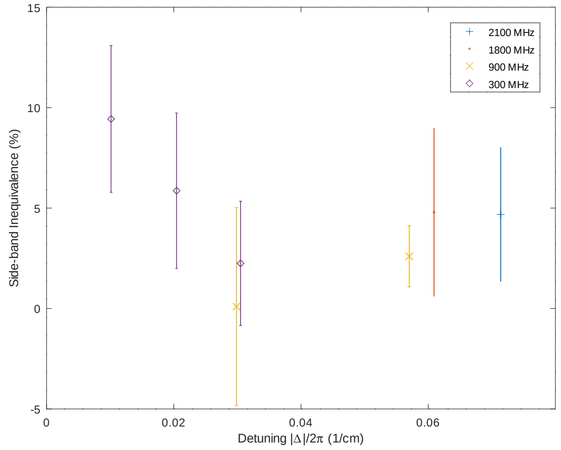

Figure S7. Side-band Inequivalence of an electrooptically modulated light computed from raw measurement data [S40].

It has to be stressed out that not every source of nonlinearity (such as cross-Kerr) or multiplying element could exhibit SI. It necessarily must be an Electrooptic medium such as externally driven $LiNbO_3$ cell or equivalent, with interaction Hamiltonian of the desired type. It remains as a subject for next studies to investigate the excessive distortion caused by this type of modulation and its implications on microwave and optical communication technologies.

S4 Brillouin Scattering

In the contexts of Brillouin Scattering (BS) and Stimulated Brillouin Scattering (SBS), SI appears very clearly which is typically easy to measure due to sharp resonances, and it could take on large magnitudes up to a few percent. One study [S42] has clearly zoomed out the Stokes and anti-Stokes resonances at various power levels, which enables to examine the variation of SI with respect to optical power to a great precision. The measured SI for illuminating optical powers of $P_{\text{op}} = \{12, 14, 20\}$mW is respectively $\bar{\delta} = \{0.059\%, 0.059\%, 0.035\%\}$ showing that an optimal operation point around $(P_{\max}, \bar{\delta}_{\max}) = (13\text{mW}, 0.059\%)$ has indeed been hit. Had the authors taken more measurement points above and below this power level, it would have been possible to reconstruct the accurate dependence. These SI numbers are accurate better within than an error margin of 0.017%.

The next article [S43] reports a very high-resolution spectrum of pump together with the blue and red side-bands in an optical fiber due to Brillouin scattering. The corresponding wavelengths are also clearly marked, enabling one to easily calculate the SI after conversion of wavelengths to frequencies. Once done, the resulting SI is $\bar{\delta} = 0.027\%$ which leans towards the red side-band as it should be. Similarly in another report [S44], the SI can be found in spectra of the transmitted light through a single-mode optical fiber $3.0\% \pm 0.21\%$, which should be evaluated with respect to the dislocated central peak.

Brillouin scattering at various angles relative to a SiOC:H/Si film has been measured and reported [S45], which shows peaks for longitudinal guided and bulk modes. Here, the SI for longitudinal bulk mode is $\bar{\delta}(\theta = 65^\circ) = 0.69\%$ and $\bar{\delta}(\theta = 25^\circ) = 0.93\%$ where θ is the illumination angle, which more or less preserves its frequency regardless of θ. The situation for guided modes is a lot more complex since it appears to be combined with the dispersion of modes like Raman scattering from Dirac materials (to be discussed next sub-section). Anyhow, one may observe approximately that $\bar{\delta}(\theta = 65^\circ) = -1.43\%$ and $\bar{\delta}(\theta = 25^\circ) = -5.01\%$ hold for longitudinal guided modes.

Brillouin scattering from surface acoustic modes of ZnO thin films grown on Si substrate is discussed in the next article [S46]. Two sets of measurements are done versus various angles of incidence $\theta = \{30^\circ, 45^\circ, 50^\circ, 60^\circ\}$ at fixed thickness of $h = 100$nm as well as different film thicknesses $h = \{20, 44, 100, 200, 320\}$nm at fixed incidence angle of $\theta = 45^\circ$. All measurements lead to definite SI considering the errorbars. In these traces, the fundamental Rayleigh wave appears in common for which it is possible to get $\bar{\delta} = \{0.99\%, 0.73\%, 3.8\%, 2.5\%\}$ as well as $\bar{\delta} = \{3.16\%, 0, 2.5\%, 6.8\%, 5.3\%\}$ within the rough error margin of $\pm 0.5\%$.

Backward Brillouin scattering may also give rise to SI. This fact may be verified on the measurements carried out in an ultra-high Q resonator [S47] which exceptionally the Stokes/anti-Stokes pair could be seen visibly. For this case, a value of $\bar{\delta} = -2.4\% \pm 0.6\%$ can be estimated, where the negative sign results from backward configuration which is equivalent to the replacement Ω by $-\Omega$.

A more recent research [S48] concerns Brillouin forward and backward scattering in various materials under two (s, p) orthogonal polarizations. For the case of microscope slab in forward scattering two resonances (L, T) can be distinguished

for each of (s, p) orthogonal polarizations, and the resulting numbers for SI mark significant deviations from zero. For $p-$polarization, we have $\bar{\delta} = \{3.2\%, 2.0\%\}$ corresponding to the two resonances while for $s-$polarization, we have $\bar{\delta} = \{4.2\%, 0.83\%\}$ all within $\pm 0.14\%$. It is instructive to examine the measurements corresponding to the forward and backward configurations which are both available for the case of cover glass. Both of the two resonances (L, T) are sharp enough in the forward configuration for both (s, p) polarizations, while in the backward configuration only one resonances can be clearly seen. The distinguishable resonance/polarization pairs here are $(L; s)$ and $(T; p)$. In the forward configuration we have $\bar{\delta}(L; p) = 0.28\%$ $\bar{\delta}(T; s) = 1.87\%$ both within $\pm 0.14\%$. Meanwhile, the SI for forward modes are $\bar{\delta}(T; p) = 1.87\%$, $\bar{\delta}(L; s) = 0.83\%$ within $\pm 0.14\%$ and for backward modes are $\bar{\delta}(T; p) = -0.37\%$, $\bar{\delta}(L; s) = -0.67\%$ within $\pm 0.09\%$. Hence, it can be confirmed well that the SI for the backward Brillouin scattering can assume negative values, as we have verified for two very different types of measurements [S47, S48].

The comprehensive set of raw measurement data of hypersonic Brillouin scattering of surface acoustic waves in bulk transparent materials has been also made available to the author [S48]. These contain a total of 12 traces shown in Fig. S8, each exhibiting 1 to 3 major resonances. By isolation of these resonances individually, and taking care of zero-calibration according to the available center resonances in the data, the SI of each resonance has been calculated one by one. There are too many plots to put here, and instead only one plot containing all SI of all traces versus frequency of each resonance is shown in Fig. S9. This shows that even taking into consideration of uncertainties, the SI of each resonance could be as large as 4% in magnitude, a remarkably large deviation from zero.

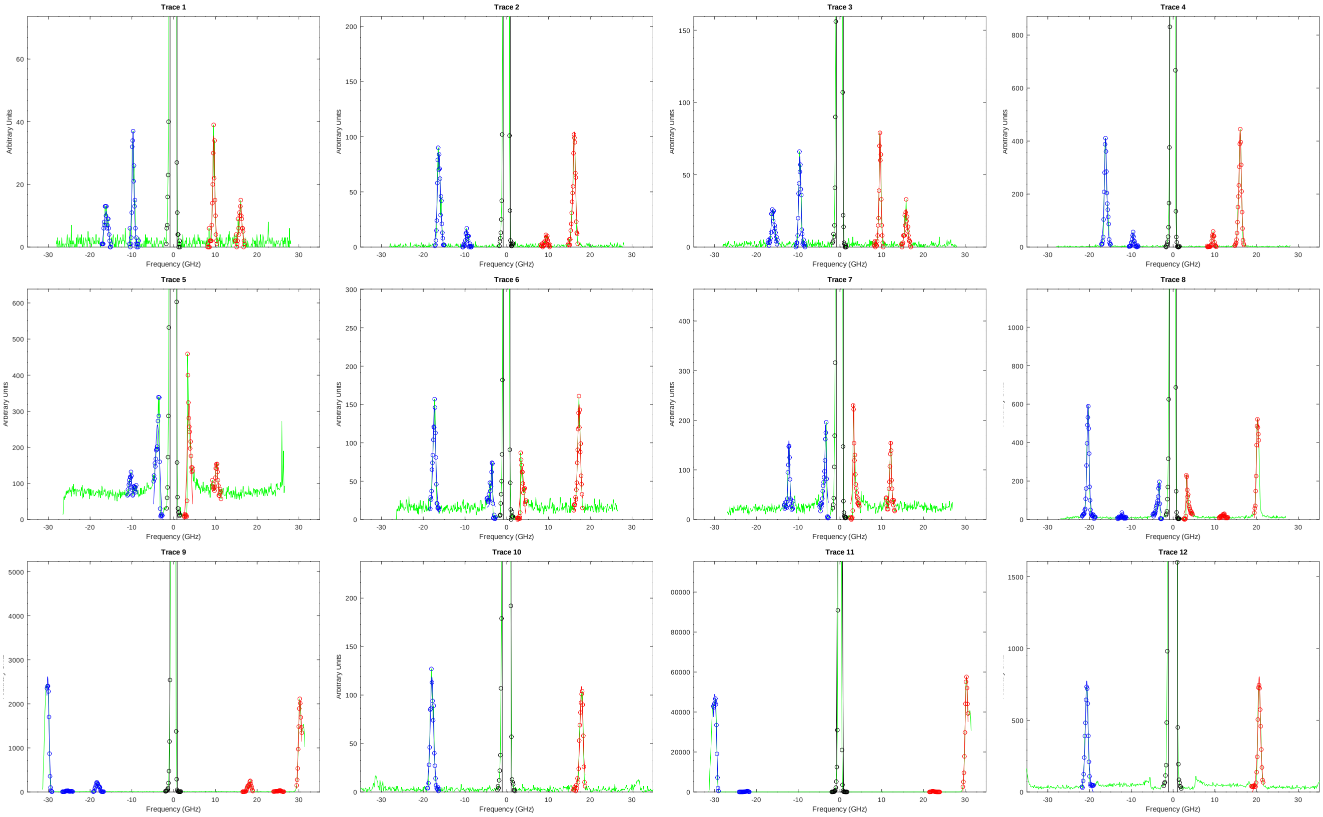

Figure S8. Hypersonic Brillouin scattering measurements for various polarizations and excitation conditions from raw measurement data [S49].

Finally, the last article in this category [S49] reports Brillouin scattering from phononic structures where accurate locations of side-bands are clearly marked throughout all figures therein, and is perfectly consistent with the positiveness of SI. The SI observed at various illumination angles for the two fabricated structures is always positive and reaches a value of 3.6%. The author was granted access to the raw measurement data, from which the figures S10,S11 were generated by making Lorentzian fits accurately to the relevant peaks.

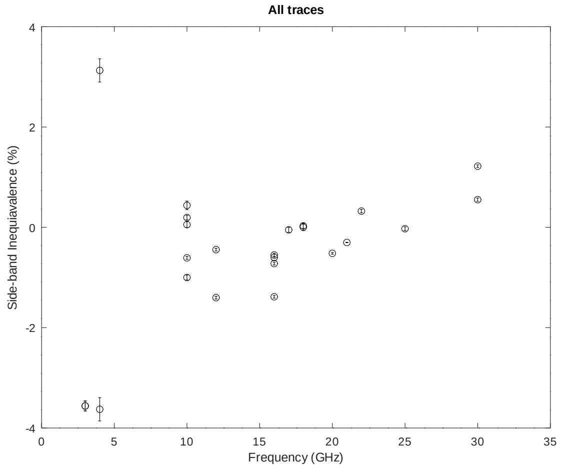

Figure S9. Collective SI points computed from raw measurement data of backward and forward Brillouin scattering of light for traces in Fig. S8, respectively with negative $\bar{\delta} < 0$ and positive $\bar{\delta} > 0$ SI for majority of points [S48].

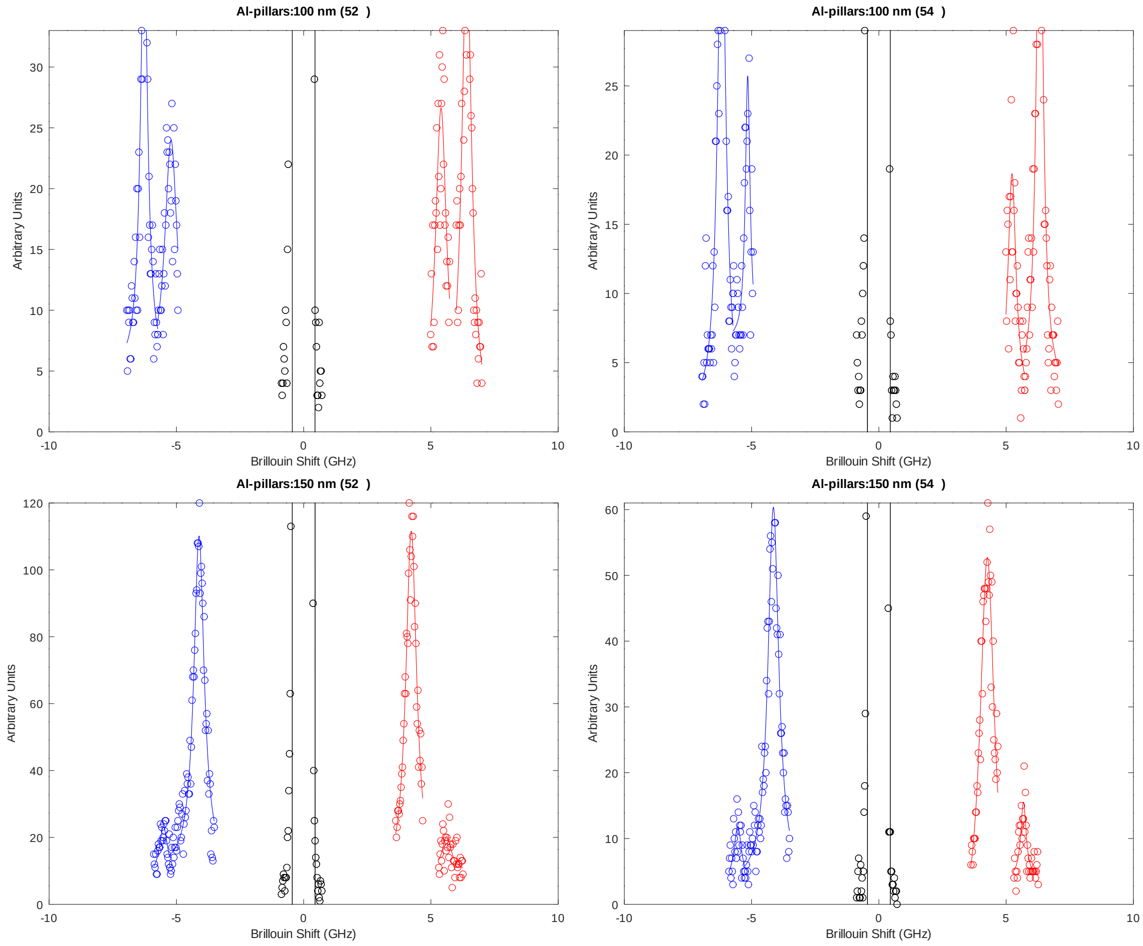

Figure S10. Lorentzian peaks for Stokes(red)/anti-Stokes(blue) pairs of Brillouin scattering measurements on Al nano pillars with different heights and illumination angles [S49].

S5 Raman Scattering

Very few researchers actually happen to have noticed the existence of an anomaly in frequency asymmetry, and clearly made a mention of it. But in majority of published works, it has gone unnoticed. Among the bulky archives of available works over the

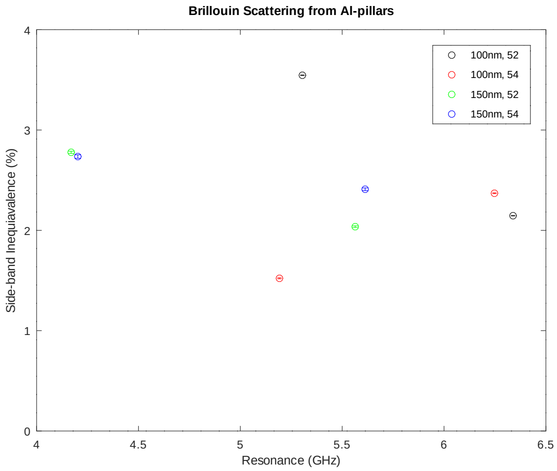

Figure S11. Side-band Inequivalences computed from raw measurement data [S49].

past decades, Raman scattering experiments provide the strongest SI of all.

However, Raman scattering is also the most complex one in the broad family of optomechanical types of interactions, where even for the simplest case of perfectly defect-free and ideal crystals, an integration over various phonon polarizations and momenta should be carried out. Hence, any non-ideal effects such as point or line defects and internal stress in piezo-electric materials could significantly alter the Raman response. For that reason, it is believed that measurements carried out on ultra-pure epitaxial cubic crystals such as Silicon (Si) are among the most reliable sources to verify the behavior of SI.

In crystalline solids, this normally reduces in effect to interaction with longitudinal optical phonons at van Hove singularities, which have nearly-identical frequencies and group velocities close to zero. For the exceptional case of Dirac materials such as graphite and graphene and metallic carbon nanotubes, the phonon dispersion together with the linear dispersion of photons can source another cause of frequency asymmetry, which normally goes in the opposite direction than that of SI. This effect however is optically linear, and does not scale with optical power.

Temperature contributions to Raman scattering are relatively significant, however, it mostly translates into enhanced optical spring effect which goes symmetrical in frequency shifts of red and blue side-bands. Since SI is all about the asymmetries, this contribution is automatically canceled out. Therefore, for graphite, the frequency asymmetry due to Anomalous Raman Phenomenon (ARP) is large enough to cause anti-Stokes shift exceed than that of Stokes resulting in $\bar{\delta} < 0$.

In gapped crystalline dielectrics such as Si, SI is strong enough to cause Stokes shift being larger than anti-Stokes, and this is particularly easily observable for the SI in characteristic Raman line 520cm^{-1} of Si.

In summary, there appears to be two competing effects which collectively contribute to frequency asymmetry in Raman scattering:

1. Side-band Inequivalence (SI): which is frequency independent but optically nonlinear, and
2. Anomalous Raman Phenomenon (ARP): which is independent of optical power but frequency dependent.

Normally, SI and ARP cause asymmetry biased towards respectively red and blue. With the exception of Dirac materials, only for which ARP has been known to exist as early as 1998 [S50], in the rest of materials, ARP could be simply neglected. This implies that effectively ARP takes over for Dirac materials, which for other crystalline solids such as Si, SI does.

The authors of the first article referring to the ARP boldly mentioned that *ARP does not arise from the error of the instrument* [S50]. That was the first major step towards realizing that Stokes and anti-Stokes pairs are not necessarily symmetrical in frequency. Initial explanations were based on the formation of doublets [S51, S52], before it was found out that it was actually the slight deviation from linear photon dispersion across the Dirac point, which could be held responsible for ARP [S53–S56].

The expression for ARP was given as $\delta = -E_s(\partial\omega_s/\partial\varepsilon_L)$ with E_s being the energy of Raman Stokes line, ω_s being the frequency of Raman shift, ε_L being energy of light photon, and the expression within the parentheses being the dispersion of Raman peak determined from electronic energy band structure. Clearly, ARP is independent of optical power and therefore is optically linear. However, it could play measureable role in Raman spectroscopy of graphene [S57–S59] and even corrections to Raman thermometry and determination of temperature, if it were to be obtained from Stokes/anti-Stokes difference [S57].

Raman spectrometry of crystalline solids contain some of the most interesting results. In a recent article, Raman scattering of the zincblende semiconductor ZnTe at various temperatures has been carried out [S60] and the raw measurement data were made available to the author. Interestingly, the longitudial (LO) and transverse (TA) optical phonons contribute differently to

the Raman spectra and the second harmonic of 2LO can also be seen. Interestingly, for most of the temperature range, both of LO and 2LO modes follow the same behavior, within a factor of 2. This is in complete agreement with the formula for the SI of second-order side-bands in quantum optomechanics in the weakly nonlinear regime [S3], and thus another way to verify the higher-order operator algebra presented therein.

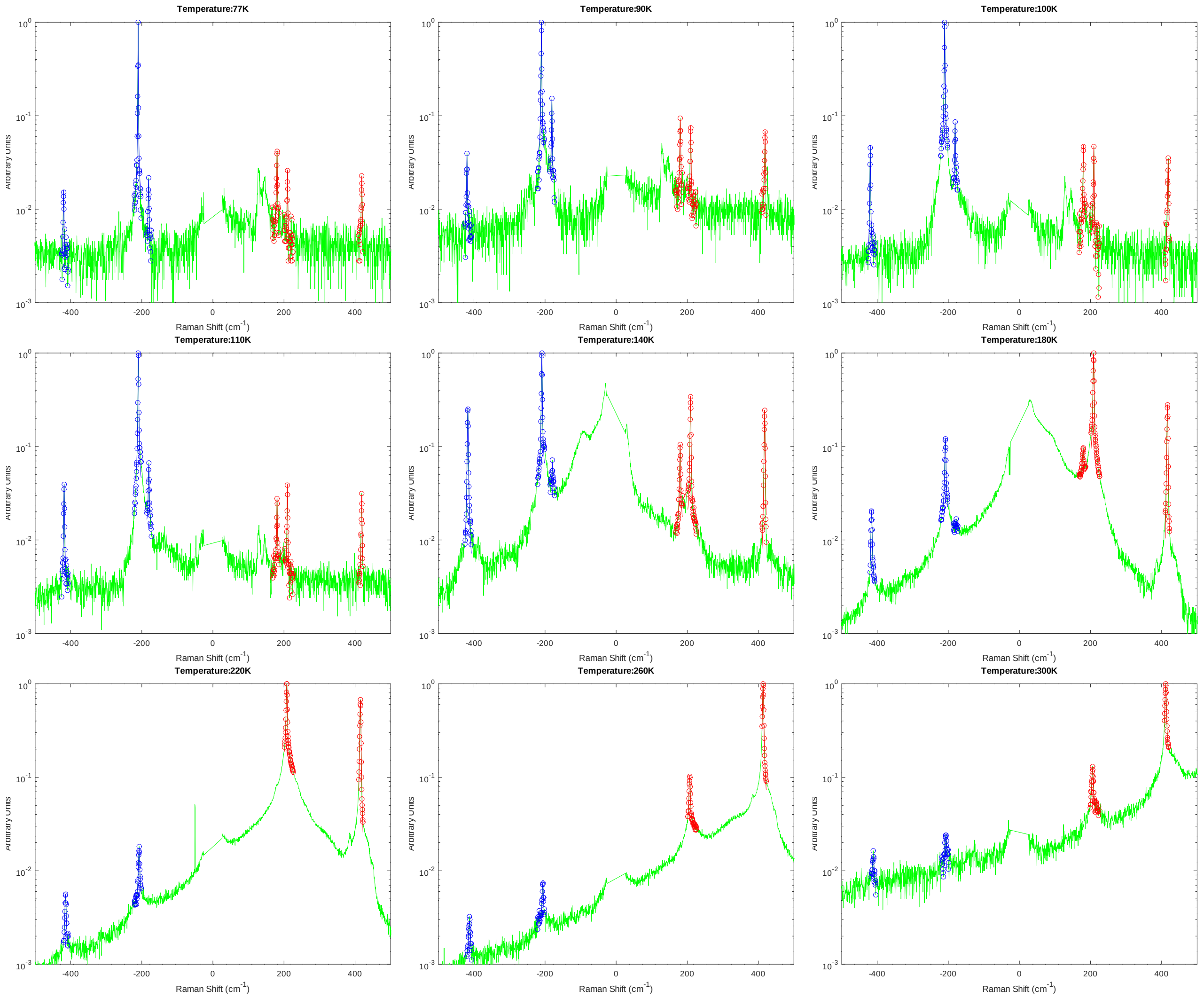

Figure S12. Lorentzian peaks for Stokes(red)/anti-Stokes(blue) pairs of Raman scattering measurements from ZnTe at various temperatures [S60].

In another study [S61], SI of three different materials, namely graphene on Si, MoS_2 on Si, and crystalline Si (with a 100nm native oxide) has been carried out at various temperatures. These are among the most extensive collection of measurements ever made available to the author in raw data format. Measurements were done using a 600gr/mm grating and for each trace both Stokes and anti-Stokes along with the central resonance were recorded at one shot. For those traces corresponding to the same material and the same temperature, trace-averages were made. Each trace exhibited a number of Raman lines which were extracted and after fitting Lorentzians and referencing to the frequency determined from the central Rayleigh peak, are shown in Fig. S14 at various temperatures. As it could be seen in Fig. S15, all SI values are positive and can be as large as 1% and decrease with Raman line frequency. Agreement to a $\bar{\delta} \propto 1/\Omega^2$ fit from (S12) is excellent.

Similar measurements were done for MoS_2 on Si samples, and the SI values at various temperatures are shown for four individual Raman lines in Fig. S16. In general, a temperature variation from room temperature to 150°C does not seem to alter the SI values appreciably.

Among these sets of measurements[S61], the most interesting is the last set done for Si, which exhibits only two resonances at 303/cm and 520/cm, and the experiment was redone and traces recorded a few times to later provide for averaging. The SI values are shown in Fig. S17 for all available traces in two forms of versus temperature and versus optical power. In Fig. S18

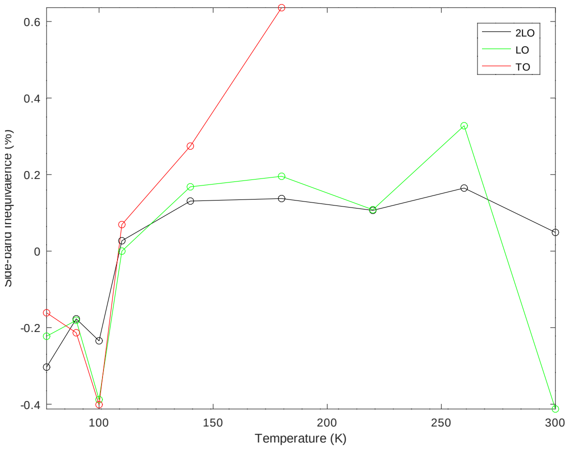

Figure S13. Side-band Inequivalences computed from raw measurement data processed in Fig. S12 [S60].

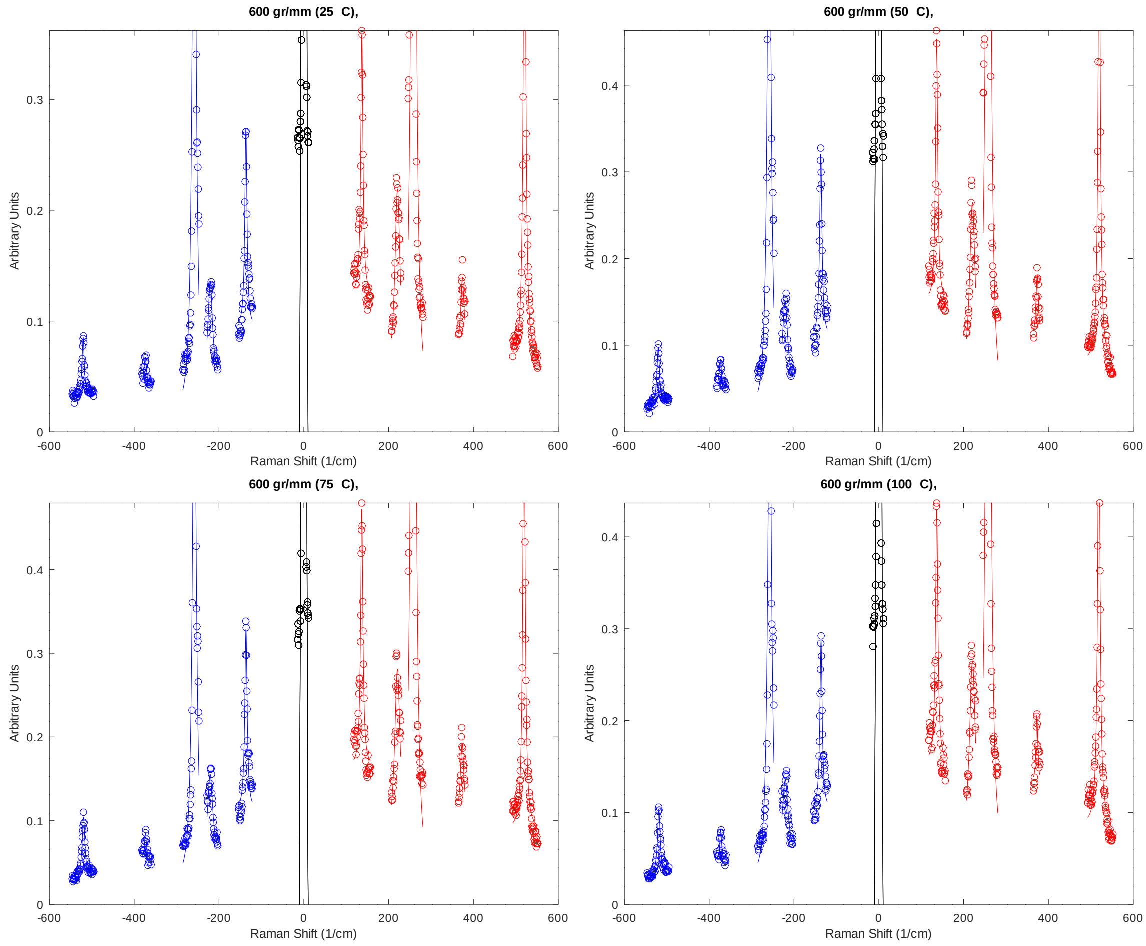

Figure S14. Lorentzian peaks for Stokes(red)/anti-Stokes(blue) pairs of Raman scattering measurements from graphene on Si substrate at various temperatures [S61].

after averaging over all traces at identical temperatures, as we learned from the previous two sets of traces.

This will make it possible to estimate a linear fit as $y = mx$ going through origin, where (x_i, y_i) samples are available. This is so since in the weakly coupled limit, SI should zero at zero optical power, behaving roughly as $\delta \approx g_0^2\bar{n}/\Omega$ [S3]. Then, the least-squares slope is simply obtained as $m = \sum(x_i y_i)/\sum x_i^2$. The ratio of obtained slopes for the two Raman lines is to be

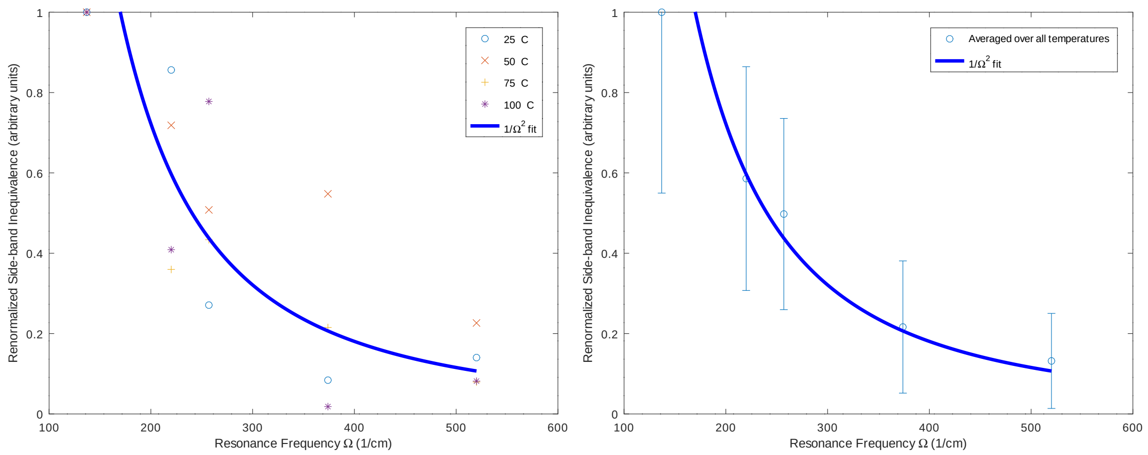

Figure S15. Side-band Inequivalences computed from raw measurement data processed in Fig. S14: SI for various Raman lines at different temperatures (left); SI for various Raman lines temperature-averaged [S61]. Note that SI values for each trace are renormalized to their maximum, to allow a simple and visible fit to (S12) which is in remarkable agreement.

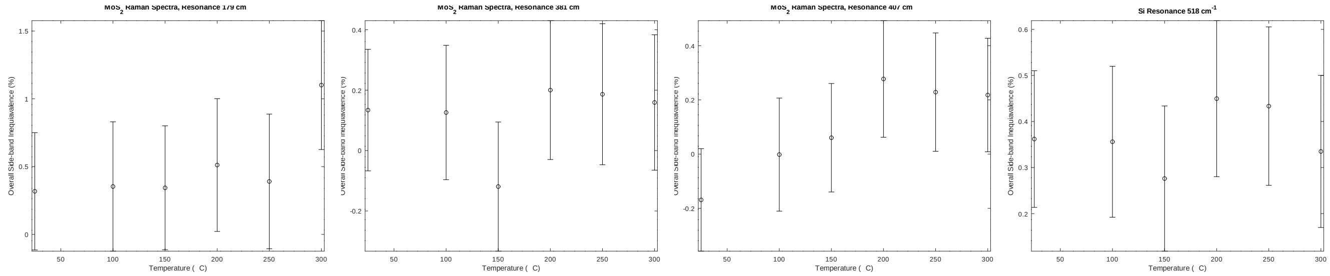

Figure S16. Variation of four identified Raman lines from MoS_2 on Si substrate with respect to substrate temperature [S61].

compared with the inverse ratio of squared frequencies and the results agree very well. Doing this will give

$$\frac{m_1}{m_2} = 0.33, \tag{S13}$$

$$\left(\frac{\Omega_1}{\Omega_2}\right)^{-2} = 0.33,$$

where subscripts 2 and 1 respectively correspond to the 303/cm and 520/cm lines.

Careful inspection of Raman scattering on two-dimensional Transition Metal Di-Chalcogenides (TMDCs) [S62, S63] exhibits significant SI for all materials at all resonances, which deserves a thorough and in-depth study.

A very recent article [S64] has reported Raman scattering study of two pervoskites $CsPbBr_3$ and $MAPbBr_3$ at various temperatures. Pervoskites are known to be very complex materials with peculiar optoelectronic and chemical properties, which are not fully understood, yet exhibit numerous Raman lines. The raw measurement data were made available to the author from which SI values of identified Raman peaks were extracted. After analysis and averaging over temperatures, the overall SI values have been found and plotted in Fig. S19. Calculated SI numbers are typically large in magnitude up to 3% and do not follow a well understood pattern. Also, Raman measurements on $NbSe_2$ [S65] for the 11-layer and bulk samples respectively show SI values of 0.46% and 4.56%, the latter being remarkably large.

In a valuable book [S66] which also contains the demonstration version of OPUS [S67] software for plotting Raman spectra, a database of measurement is supplied out of which traces of four materials contain both Stokes and anti-Stokes side-bands. Typically, these reference measurements are averaged over hundreds of Raman scattering to remove background noise and improve the visibility of resonances. To a great extent, these measurements seem reliable and furthermore, contain the central Rayleigh resonance which allows accurate zero centering. These four mentioned substances are Ceramide, heavy Ethanol, Stearic Acid, and Sulphur. With the exception of first resonance of Stearic Acid, all these exhibit remarkably strong and positive SI, as shown in Fig. S20.

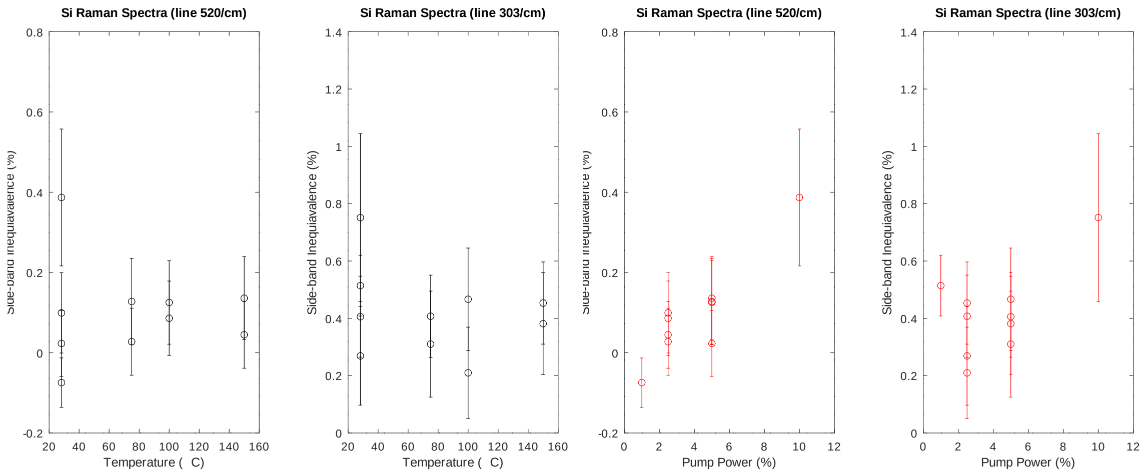

Figure S17. Variation of SI for two characteristic lines of Si versus temperature (left in black) and versus optical power (right in red) for available traces [S61].

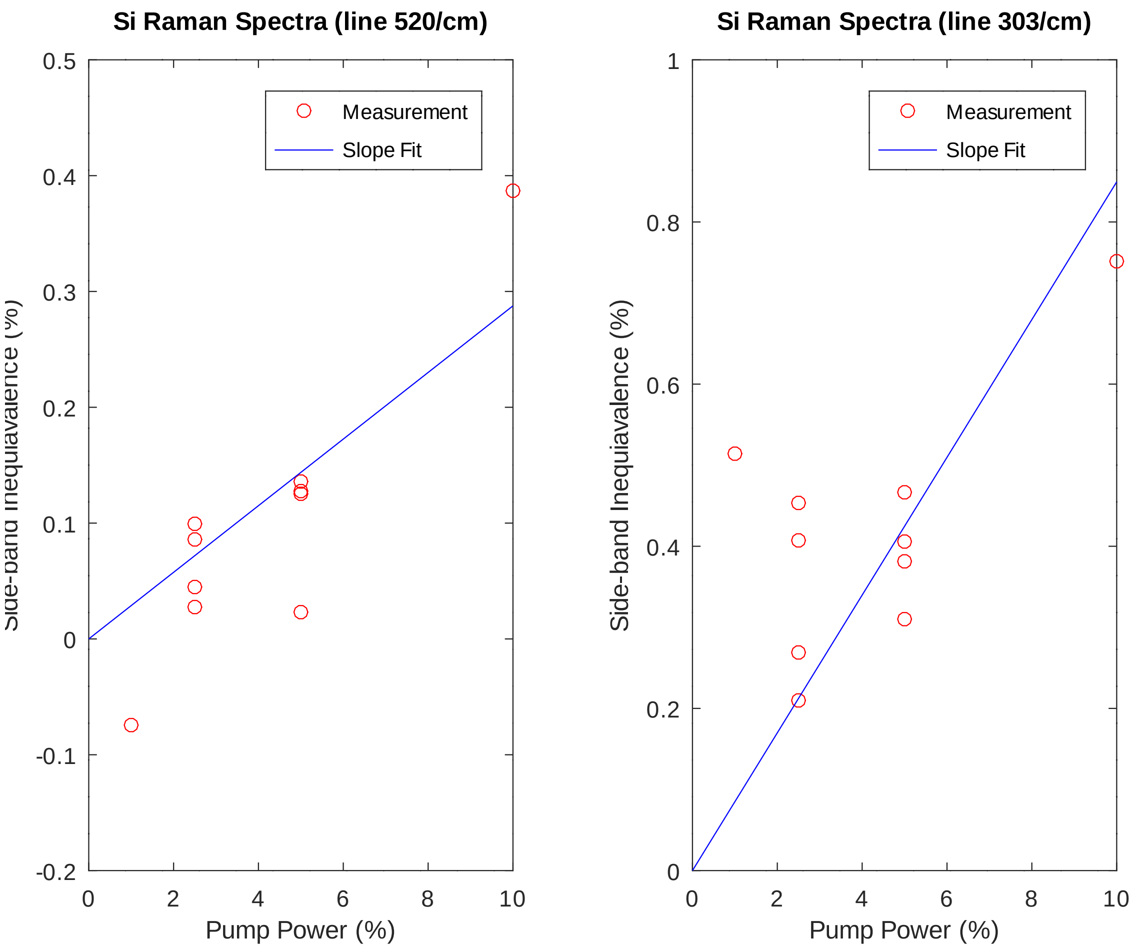

Figure S18. Variation of SI versus optical power for the two major lines at 303/cm and 520/cm [S61].

The Coherent company has developed a new technology for precision remote Raman analysis of various materials, many examples of which are publicized in their newest brochure [S68]. It is very interesting to take a look at the calculated SI values from their available data. All these very different substances, (1) Carbamazepine (a common prescription for epilepsy and other

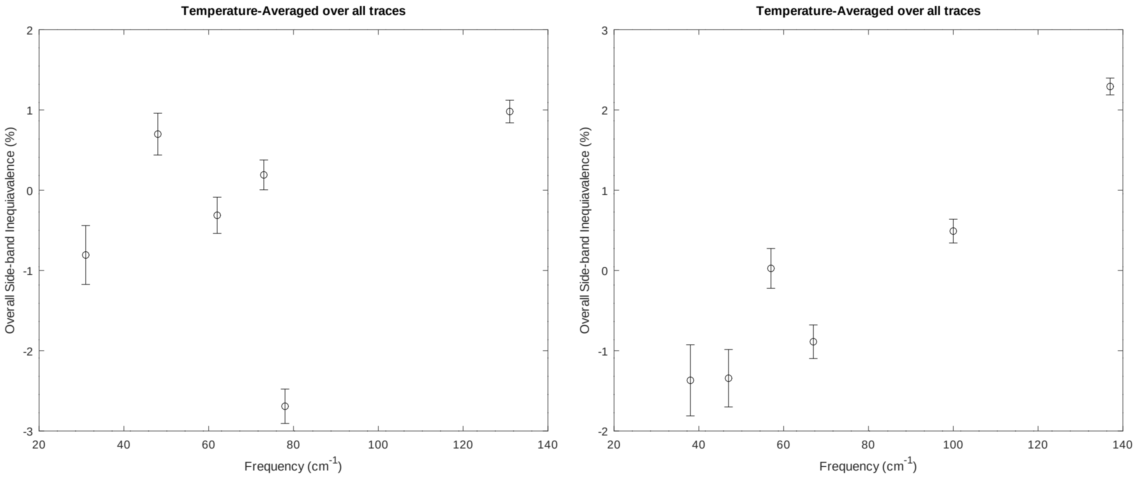

Figure S19. Variation of SI after temperature averaging of identified Raman lines for two pervoskites $CsPbBr_3$ and $MAPbBr_3$ [S64] from raw measurement data.

neurologic disorders), (2) Carbamazepine hydrate, (3) Crystallized Caffeine (the extract and essential ingredient of coffee), and (4) Sulphur in three different configurations (α: crystalline; β: amorphous; λ: liquid) exhibit large SI numbers up for many Raman lines to a few percent. In all four studied cases, SI is positive and significantly larger than zero.

In the end, Crystallized Coffee exhibits five Raman lines at 42.6/cm, 61.5/cm, 105.4/cm, 122.6/cm, and 180.1/cm, with SI values respectively equal to 2.06%, 1.43%, 0.833%, 0.717%, and 0.488%. Surprisingly or not, these very well fit to (S12) with a total optomechanical decay rate of $\gamma = 2\pi \times 25\text{cm}^{-1}$, reminding Alfréd Rényi and legendary Paul Erdös who believed [S69] "*A mathematician is a machine for turning coffee into theorems*."

Acknowledgement

The author wishes to sincerely thank many colleagues and scientists as well as their teams around the world who generously either supplied their valuable measurements or shared their experimental equipment to provide the basis of this analysis. In particular, the author is highly indebted to Ms. Prof. Aleksandra Trzaskowska at Adam Mickiewicz University, Prof. Eric Pop and Dr. Sam Vaziri at Stanford University, Prof. Eilam Yalon at Technion, Prof. Mikkel Andersen at University of Otago, Prof. Rafael J. Jimménez Riobóo at Instituto de Ciencia de Materiales de Madrid, Prof. Louis E. Brus at Columbia University, Prof. Christian F. Roos at University of Innsbruck, Prof. Jun Zhang at Chinese Academy of Sciences, Prof. Dzmitry Matsukevich at National University of Singapore, Prof. Lilin Yi at Shanghai Jiao Tong University, Prof. Jeremy Baumberg at University of Cambridge, Prof. Shin-chi Zaitsu at Kyushu University, and Prof. Huihui Lu at Jinan University for providing access to raw measurement data. Discussions with Dr. Andrey Matsko at OE Waves as well as Prof. Alexander Tagantsev and members of the Laboratory of Photonics and Quantum Measurements at École Polytechnique Fédérale de Lausanne is much appreciated.

References

[S1] Laplace, P. S. *Théorie Analytique des Probabilités* (Paris, Courcier, 1812).

[S2] Aspelmeyer, M., Kippenberg, T.J. & Marquardt, F. Cavity optomechanics. *Rev. Mod. Phys.* **86**, 1391 (2014).

[S3] Khorasani, S. Method of higher-order operators for quantum optomechanics. *Sci. Rep.* **8**, 11566 (2018).

[S4] Lachance-Quirion, S., Tabuchi, Y., Gloppe, A., Usami, K. & Nakamura, Y. Hybrid quantum systems based on magnonics. *arxiv*, 1902.03024 (2019).

[S5] Zhang, S., Zou, C.-L., Jiang, L. & Tang, H. X. Cavity magnomechanics. *Sci. Adv.* **2**, e1501286 (2016).

[S6] Khorasani, S. Momentum-field interactions beyond standard quadratic optomechanics. In *Quantum Mechanics: Theory, Analysis, and Applications* (ed. Arbab, A. I.) 1–17 (Nova Science Publishers, 2018).

[S7] Otterstrom, N. T., Behunin, R. O., Kittlaus, E. A. & Rakich, P. T. Optomechanical cooling in a continuous system. *Phys. Rev. X* **8**, 041034 (2018).

[S8] Schliesser, A., Rivière, R., Anetsberger, G., Arcizet, O. & Kippenberg, T. J. Resolved-sideband cooling of a micromechanical oscillator. *Nat. Phys.* **4**, 415 (2008).

[S9] Sudhir, V., Wilson, D. J., Schilling, R., Schütz, H., Fedorov, S. A., Ghadimi, A. H., Nunnenkamp, A. & Kippenberg,

T. J. Appearance and disappearance of quantum correlations in measurement-based feedback control of a mechanical oscillator. *Phys. Rev. X* **7**, 011001 (2017).

[S10] Underwood, M., Mason, D., Lee, D., Xu, H., Jiang, L., Shkarin, A. B., Børkje, K., Girvin, S. M. & Harris, J. G. E. Measurement of the motional sidebands of a nanogram-scale oscillator in the quantum regime. *Phys. Rev. A* **92**, 061801(R) (2015).

[S11] Golter, D. A., Oo, T., Amezcua, M., Stewart, K. A. & Wang, H. Optomechanical quantum control of a Nitrogen-vacancy center in diamond. *Phys. Rev. Lett.* **116**, 143602 (2016).

[S12] Sompet, P., Fung, Y. H., Schwartz, E., Hunter, M. D. J., Phrompao, J. & Andersen, M. F. Zeeman-insensitive cooling of a single atom to its two-dimensional motional ground state in tightly focused optical tweezers. *Phys. Rev. A* **95**, 031403(R) (2017).

[S13] Kaufman, A. M., Lester, B. J. & Regal, C. A. Cooling a single atom in an optical tweezer to its quantum ground state. *Phys. Rev. X* **2**, 041014 (2012).

[S14] Shomroni, I., Qiu, L., Malz, D., Nunnenkamp, A. & Kippenberg, T. J. Optical backaction-evading measurement of a mechanical oscillator. *Nat. Commun.* **10**, 2086 (2019).

[S15] Shomroni, I., Qiu, L., Malz, D., Nunnenkamp, A. & Kippenberg, T. J. Optical backaction-evading measurement of a mechanical oscillator. [Data set] *Zenodo*, doi:10.5281/zenodo.2563797 (2019).

[S16] Akhmediev, N. N., Eleonskii, V. M. & Kulagin, N. E. *Theor. Math. Phys.* **72**, 809 (1987).

[S17] Xiong, H. & Wu, Y. Optomechanical Akhmediev breathers. *Laser Photon. Rev.* **12**, 1700305 (2018).

[S18] Chuah, B. L., Lewty, N. C., Cazan, R. & Barrett, M. D. Detection of ion micromotion in a linear Paul trap with a high finesse cavity. *Opt. Express* **21**, 10632 (2013).

[S19] Roos, C., Zeiger, T., Rohde, H., Nägerl, H. C., Eschner, J., Leibfried, D., Schmidt-Kaler, F. & Blatt, R. Quantum state engineering on an optical transition and decoherence in a Paul trap. *Phys. Rev. Lett.* **83**, 4713 (1999).

[S20] Schulz, S. A., Poschinger, U., Ziesel, F. & Schmidt-Kaler, F. Sideband cooling and coherent dynamics in a microchip multi-segmented ion trap. *New J. Phys.* **10**, 045007 (2008).

[S21] Wilpers, G., See, P., Gill, P. & Sinclair, A. G. A monolithic array of three-dimensional ion traps fabricated with conventional semiconductor technology. *Nat. Nanotech.* **7**, 572 (2012).

[S22] Guise, N. D., Fallek, S. D., Stevens, K. E., Brown, K. R., Volin, C., Harter, A. W., Amini, J. M., Higashi, R. E., Lu, E. T., Chanhvongsak, H. M., Nguyen, T. A., Marcus, M. S., Ohnstein, T. R. & Youngner, D. W. Ball-grid array architecture for microfabricated ion traps. *J. Appl. Phys.* **117**, 174901 (2015).

[S23] An, D., Matthiesen, C., Abdelrahman, A., Berlin-Udi, M., Gorman, D., Möller, S., Urban, E. & Häffner, H. Surface trap with dc-tunable ion-electrode distance. *Rev. Sci. Instrum.* **89**, 093102 (2018).

[S24] Doret, S. C., Amini, J. M., Wright, K., Volin, C., Killian, T., Ozakin, A., Denison, D., Hayden, H., Pai, C.-S. & Slusher, R. E. Controlling trapping potentials and stray electric fields in a microfabricated ion trap through design and compensation. *New J. Phys.* **14**, 073012 (2012).

[S25] Nägerl, H. C., Roos, C., Leibfried, D., Rohde, H., Thalhammer, G., Eschner, J., Schmidt-Kaler, F. & Blatt, R. Investigating a qubit candidate: Spectroscopy on the $S_{1/2}$ to $D_{5/2}$ transition of a trapped calcium ion in a linear Paul trap. *Phys. Rev. A* **61**, 023405 (2000).

[S26] Hayes, D., Matsukevich, D. N., Maunz, P., Hucul, D., Quraishi, Q., Olmschenk, S., Campbell, W., Mizrahi, J., Senko, C. & Monroe, C. Entanglement of atomic qubits using an optical frequency comb. *Phys. Rev. Lett.* **104**, 140501 (2010).

[S27] Rohde, H., Gulde, S. T., Roos, C. F., Barton, P. A., Leibfried, D., Eschner, J., Schmidt-Kaler, F. & Blatt, R. Sympathetic ground-state cooling and coherent manipulation with two-ion crystals. *J Opt. B: Quantum Semiclass. Opt.* **3**, S34 (2001).

[S28] Deslauriers, L., Haljan, P. C., Lee, P. J., Brickman, K.-A., Blinov, B. B., Madsen, M. J. & Monroe, C. Zero-point cooling and low heating of trapped $^{111}Cd^{+}$ ions. *Phys. Rev. A* **70**, 043408 (2004).

[S29] Neuzner, A., Dürr, S., Körber, M., Ritter, S. & Rempe, G. Increased dimensionality of Raman cooling in a slightly nonorthogonal optical lattice. *Phys. Rev. A* **98**, 013401 (2018).

[S30] Neuzner, A. Resonance fluorescence of an atom pair in an optical resonator. *PhD Thesis*, Technische Universität München (2016), Chap. 4.2.

[S31] Lechner, R., Maier, C., Hempel, C., Jurcevic, P., Lanyon, B. P., Monz, T., Brownnutt, M., Blatt, R. & Roos, C. F. Electromagnetically-induced-transparency ground-state cooling of long ion strings. *Phys. Rev. A* **93**, 053401 (2016).

[S32] Ding, S., Loh, H., Hablutzel, R., Gao, M., Maslennikov, G. & Matsukevich, D. Microwave control of trapped-ion motion assisted by a running optical lattice. *Phys. Rev. Lett.* **113**, 073002 (2014).

[S33] Xu, Z. T., Yuan, W. H., Zeng, X. Y., Che, H., Shi, X. H., Deng, K., Zhang J. & Lu, Z. H. Recent progress on the $^{27}Al^{+}$ ion optical clock. *J. Phys.: Conf. Ser.* **723**, 012026 (2016).

[S34] Brahms, N., Botter, T., Schreppler, S., Brooks, D. C. W. & Stamper-Kurn, D. M. Optical detection of the quantization of collective atomic motion. *Phys. Rev. Lett.* **108**, 133601 (2012).

[S35] Niklès, M., Thévenaz, L. & Robert, P. A. Brillouin gain spectrum characterization in single-mode optical fibers. *J. Lightwave Technol.* **15**, 1842 (1997).
[S36] Niklès, M., Thévenaz, L. & Robert, P. A. Simple distributed fiber sensor based on Brillouin gain spectrum analysis. *Opt. Lett.* **21**, 758 (1996).
[S37] Caponi, S., Dionigi, M., Fioretto, S., Mattarelli, M., Palmieri, L. & Socino, G. Electro-optic modulator for high resolution Brillouin scattering measurements. *Rev. Sci. Instrum.* **72**, 198 (2001).
[S38] Stiller, B. Brillouin scattering in photonic crystal fiber: from fundamentals to fiber optic sensors. *PhD Thesis*, Université de Franche-Comté (2011).
[S39] Shi, M., Yi, L., Wei, W. & Hu, W. Generation and phase noise analysis of a wide optoelectronic oscillator with ultra-high resolution based on stimulated Brillouin scattering. *Opt. Express* **26**, 16113 (2018).
[S40] Lu, H., Qiu, W., Guyot, C., Ulliac, G., Merolla, J.-M., Baida, F., Bernal, M.-P., Optical and RF characterization of a Lithium Niobate photonic crystal modulator. *IEEE Photonics Technol. Lett.* **26**, 1332 (2014).
[S41] Zaitsu, S.-I. Izaki, H., Tsuchiya, T. & Imasaka, T. Continuous-wave phase-matched molecular optical modulator. *Sci. Rep.* **6**, 20908 (2016).
[S42] Shin, H., Qiu, W., Jarecki, R., Cox, J. A., Olsson III, R. H., Starbuck, A., Wang, Z. & Rakich, P. T. Tailorable stimulated Brillouin scattering in nanoscale silicon waveguides. *Nat. Commun.* **4**, 1944 (2013).
[S43] Mridha, M. K., Novoa, D. & Russell, P. St. J. Dominance of backward stimulated Raman scattering in gas-filled hollow-core photonic crystal fibers. *Optica* **5**, 570 (2018).
[S44] Kang, M. S., Nazarkin, A., Brenn, A. & Russell, P. St. J. Tightly trapped acoustic phonons in photonic crystal fibres as highly nonlinear artificial Raman oscillators. *Nat. Phys.* **5**, 276 (2009).
[S45] Carlotti, G., Chérault, N., Casanova, N., Goldberg, C. & Socino, G. Elastic constants of low-k and barrier dielectric films measured by Brillouin light scattering. *Thin Solid Films* **493**, 175 (2005).
[S46] Carlotti, G., Fioretto, D., Palmieri, L., Socino, G., Verdini, L. & Verona, E. Brillouin scattering by surface acoustic modes for elastic characterization of ZnO films. *IEEE Trans. Ultrason. Ferroelectr. Freq. Control* **38**, 56 (1991).
[S47] Lin, G., Diallo, S., Dudley, J. M. & Chembo, Y. K. Universal nonlinear scattering in ultra-high Q whispering gallery-mode resonators. *Opt. Express* **24**, 14880 (2016).
[S48] Jiménez Riobóo, R. J., Sánchez-Sánchez, A. & Prieto, C. Optical find of hypersonic surface acoustic waves in bulk transparent materials. *Phys. Rev. B* **94**, 014313 (2016).
[S49] Graczykowski, B., Mielcarek, S., Trzaskowska, A., Sarkar, J., Hakonen, P. & Mroz, B. Tuning of a hypersonic surface phononic band gap using a nanoscale two-dimensional lattice of pillars. *Phys. Rev. B* **86**, 085426 (2012).
[S50] Tan, P.-H., Deng, Y.-M. & Zhao, Q. Temperature-dependent Raman spectra and anomalous Raman phenomenon of highly oriented pyrolytic graphite. *Phys. Rev. B* **58**, 5435 (1998).
[S51] Thomsen, C. & Reich, S. Double resonant Raman scattering in graphite. *Phys. Rev. Lett.* **85**, 5214 (2000).
[S52] Brown, S. D. M., Corio, P., Marucci, A., Dresselhaus, M. S., Pimenta, M. A. & Kneipp, K. Anti-Stokes Raman spectra of single-walled carbon nanotubes. *Phys. Rev. B* **61**, R5137(R) (2000).
[S53] Tan, P.-H., Hu, C.-Y., Dong, J., Shen, W.-C. & Zhang, B.-F. Polarization properties, high-order Raman spectra, and frequency asymmetry between Stokes and anti-Stokes scattering of Raman modes in a graphite whisker. *Phys. Rev. B* **64**, 214301 (2001).
[S54] Tan, P. H., An, L., Liu, L.-Q., Guo, Z.-X., Czerw, R., Carroll, D. L., Ajayan, P. M., Zhang, N. & Guo, H. L. Probing the phonon dispersion relations of graphite from the double-resonance process of Stokes and anti-Stokes Raman scatterings in multiwalled carbon nanotubes. *Phys. Rev. B* **66**, 245410 (2002).
[S55] Zólyomi, V. & Kürti, J. Calculating the discrepancy between the Stokes and anti-Stokes Raman D band of carbon nanotubes using double resonance theory. *Phys. Rev. B* **66**, 073418 (2002).
[S56] Tan, P.-H., Hu, C.-Y., Dong, J. & Shen, W.-C. Double resonance Raman scattering of second-order Raman modes from an individual graphite whisker. *Physica E* **37**, 93 (2007).
[S57] Ferrari, A. C. & Basko, D. M. Raman spectroscopy as a versatile tool for studying the properties of graphene. *Nat. Nanotech.* **8**, 235 (2013).
[S58] Romagnoli, M., Sorianello, V., Midrio, M., Koppens, F. H. L., Huyghebaert, C., Neumaier, D., Galli, P., Templ, W., D'Errico, A. & Ferrari, A. C. Graphene-based integrated photonics for next-generation datacom and telecom. *Nat. Rev. Mater.* **3**, 392 (2018).
[S59] Ferrante, C., Virga, A., Benfatto, L., Martinati, M., De Fazio, D., Sassi, U., Fasolato, C., Ott, A. K., Postorino, P., Yoon, D., Cerullo, G., Mauri, F., Ferrari, A. C. & Scopigno, T. Raman spectroscopy of graphene under ultrafast laser excitation. *Nat. Commun.* **9**, 308 (2018).
[S60] Zhang, J., Zhang, Q., Wang, X., Kwek, L.-C. & Xiong, Q. Resolved-sideband Raman cooling of an optical phonon in semiconductor materials. *Nat. Photon.* **10**, 600 (2016).

[S61] Yalon, E., Deshmukh, S., Rojo, M. M., Lian, F., Neumann, C. M., Xiong, F. & Pop, E. Spatially resolved thermometry of resistive memory devices. *Sci. Rep.* **7**, 15360 (2017).

[S62] Zhang, X., Han, W. P., Wu, J. B., Milana, S., Lu, Y., Li, Q. Q., Ferrari, A. C. & Tan, P. H. Raman spectroscopy of shear and layer breathing modes in multilayer MoS_2. *Phys. Rev. B* **87**, 115413 (2013).

[S63] O'Brien, M., McEvoy, N., Hanlon, D., Hallam, T., Coleman, J. N. & Duesberg, G. S. Mapping of low-frequency Raman modes in CVD-grown transition metal dichalcogenides: Layer number, stacking orientation and resonant effects. *Sci. Rep.* **6**, 19476 (2016).

[S64] Guo, Y., Yaffe, O., Hull, T. D., Owen, J. S., Reichman, D. R. & Brus, L. E. Dynamic emission Stokes shift and liquid-like dielectric solvation of band edge carriers in lead-halide perovskites. *Nat. Commun.* **10**, 1175 (2019).

[S65] Xi, X., Zhao, L., Wang, Z., Berger, H., Forró, L., Shan, J. & Mak, K. F. Strongly enhanced charge-density-wave order in monolayer $NbSe_2$. *Nat. Nanotech.* **10**, 765 (2015).

[S66] Wartewig, S. *IR and Raman Spectroscopy: Fundamental Processing* (Wiley-VCH Verlag, Weinheim, 2005).

[S67] Bruker, Co., *OPUS Spectroscopy Software*. http://www.bruker.com/.

[S68] Coherent, Inc., *Extending Raman into the THz Domain: Delivering Both Chemical Composition and Structural Information in a Single Measurement* (Santa Clara, 2019).

[S69] Suzuki, J. *A History of Mathematics* (Prentice-Hall, 2002), p. 731.

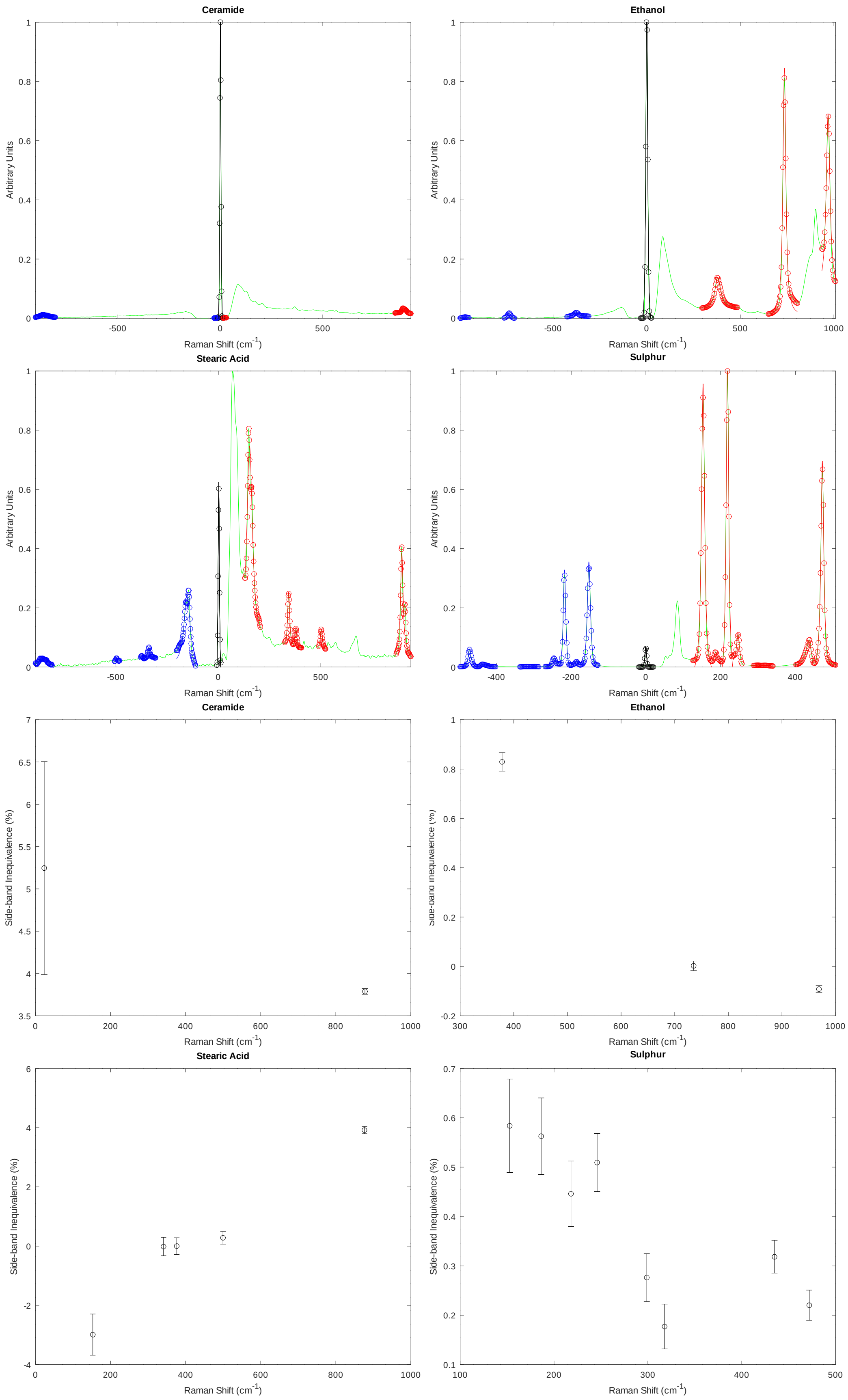

Figure S20. Raman spectra (top rows) and corresponding SI values (bottom rows) from raw measurement data [S65] for Ceramide, Ethanol, Stearic Acid, and Sulphur.

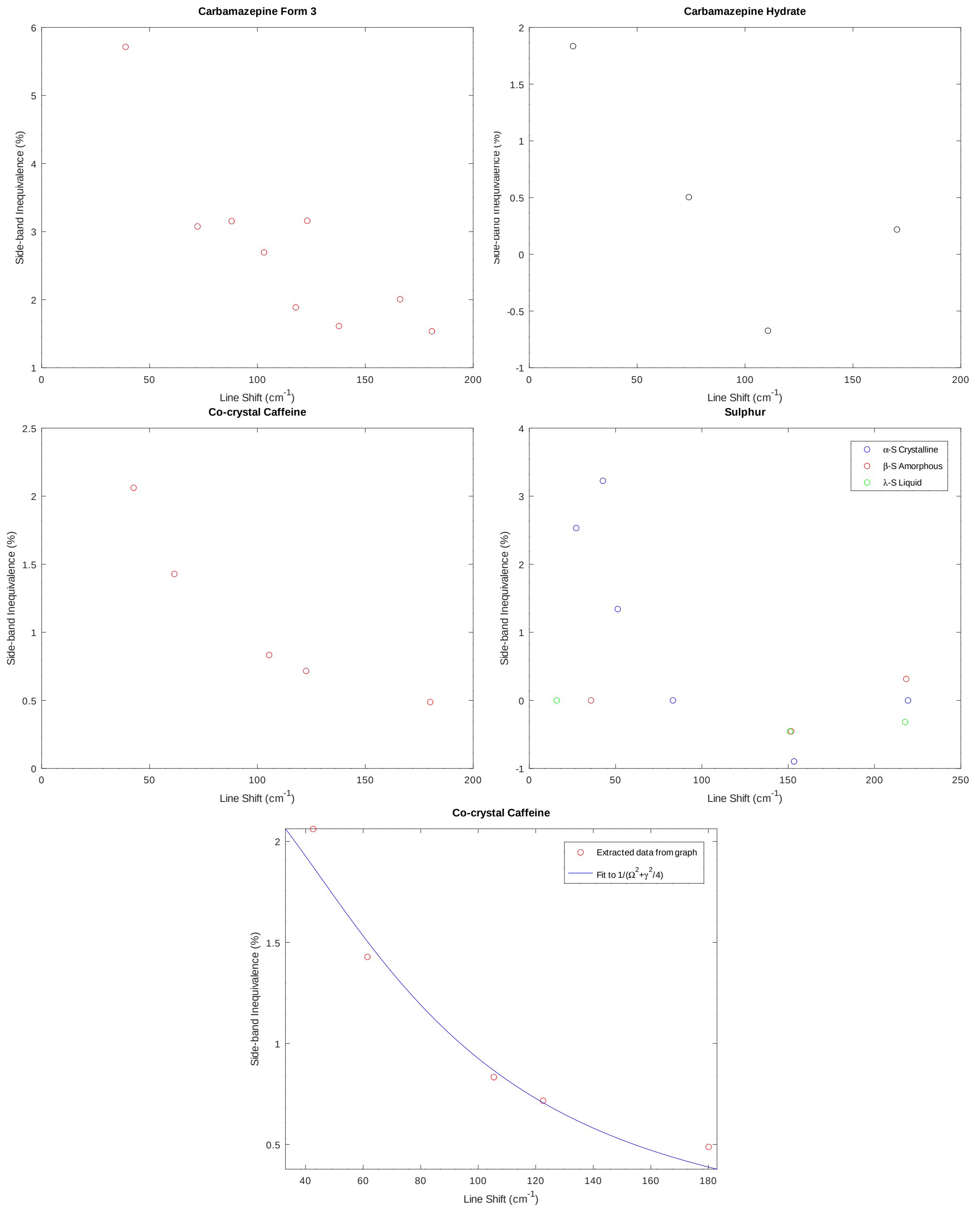

Figure S21. Calculated SI values extracted for four very different materials using a new THz/Raman scattering technology: (top-left) Carbamazepine, a common medication for epilepsy and neurologic disorders; (top-right) Carbamazepine hydrate; (middle-left) Crystallized Caffeine, the extract of beloved hot drink coffee (middle-right) Sulphur in three different configurations (α: crystalline; β: amorphous; λ: liquid); (bottom) SI for Crystallized Caffeine extracted from measured graph, with fitting to (S12) with $\gamma = 157.1\text{cm}^{-1}$ [S68].
